# Multi-omic profiling and pathways related to changes in liver histology after Roux-en-Y gastric bypass: a longitudinal study

**DOI:** 10.1016/j.ebiom.2026.106498

**Published:** 2026-09-30

**Authors:** Yasaman Ghorbani, Katherine J.P. Schwenger, Heather Maughan, Anastasia Teterina, Wendy Lou, Elena M. Comelli, Sandra E. Fischer, Timothy D. Jackson, Allan Okrainec, Johane P. Allard

**Affiliations:** a Toronto General Hospital, University Health Network, Toronto, Ontario, Canada; b Institute of Medical Science, University of Toronto, Toronto, Ontario, Canada; c National Coalition of Independent Scholars, Brattleboro, Vermont, US; d Dalla Lana School of Public Health, University of Toronto, Toronto, Ontario, Canada; e Department of Nutritional Sciences, University of Toronto, Toronto, Ontario, Canada; f Department of Laboratory Medicine and Pathobiology, University of Toronto, Toronto, Ontario, Canada; g Division of General Surgery, University of Toronto, Toronto, Ontario, Canada; h Division of General Surgery, Toronto Western Hospital, University Health Network, Toronto, Ontario, Canada; i Department of Medicine, University of Toronto, Toronto, Ontario, Canada

**Keywords:** Metabolic dysfunction-associated steatotic liver disease, Roux-en-Y gastric bypass, Mitochondrial oxidation, Transcriptome, Metabolome, Microbiome

## Abstract

**Background:**

Roux-en-Y gastric bypass (RYGB) improves metabolic dysfunction-associated steatotic liver disease (MASLD). However, the impact of RYGB on hepatic transcriptome, faecal microbiome and serum/faecal metabolome remain understudied. Our objective was to investigate the change in these omics and their relationships with changes in liver histology.

**Methods:**

In this prospective cohort study, patients undergoing RYGB were recruited between 2013 and 2020 and followed for 12 months. Anthropometrics, biochemistry, hepatic transcriptome, faecal microbiome (shotgun metagenomics) and serum/faecal metabolomes were measured. Liver histology and NAFLD Score (NAS) were assessed.

**Findings:**

Thirty-eight patients completed the study. Anthropometrics, biochemical and histological parameters improved post-RYGB (p < 0.05). Hepatic transcriptome analysis revealed a co-expression module enriched in fatty acid metabolism which correlated with changes in NAS post-RYGB (ρ = 0.38, p = 0.019). The core enrichment genes in this pathway were involved in mitochondrial and peroxisomal β-oxidation (ACADVL, ACOX1, and EHHADH) and the tricarboxylic acid (TCA) cycle (SUCLG2, SDHC, and SERINC1). There was an increase in TCA cycle gene expression associated with the resolution of ballooning, while upregulation of β-oxidation genes correlated with less reduction in NAS, ballooning, and inflammation. Metabolomic changes related to the identified co-expression module and pathways reveal a significant increase in faecal acylcarnitines, likely due to malabsorption from RYGB, with a significant reduction in circulating acylcarnitines which correlated positively with SUCLG2 expression and resolution of ballooning. Additionally, the increase in faecal acylcarnitines positively correlated with the bacterial species utilising acylcarnitines. In network analysis, ballooning of hepatocytes was associated with faecal/serum acylcarnitines and TCA metabolites while SUCLG2 was the hub gene associated with these changes.

**Interpretation:**

These findings provide insight on how post-RYGB changes in the transcriptome, metabolome, and microbiome could be associated with improvement in liver histology and may inform the development of future strategies for MASLD management.

**Funding:**

10.13039/501100000024Canadian Institutes of Health Research and 10.13039/100007827American College of Gastroenterology.

**Research in context**

**Evidence before this study**

We searched PubMed from database inception to February 2026 using combinations of the terms “metabolic dysfunction-associated steatotic liver disease”, “MASLD”, “non-alcoholic fatty liver disease”, “NAFLD”, “metabolic dysfunction-associated steatohepatitis”, “MASH”, “non-alcoholic steatohepatitis”, “NASH”, “Roux-en-Y gastric bypass”, “RYGB”, “bariatric surgery”, “hepatic transcriptome”, “gene expression”, “microbiome”, and “metabolome”. The prevalence of MASLD is increasing and effective therapeutic options remain limited. RYGB is an effective weight loss surgery which can contribute to MASLD improvement. However, the molecular mechanism(s) underlying these improvements is not fully understood. Previous studies demonstrated that MASLD have altered hepatic transcriptome, intestinal microbiome and metabolome compared to healthy individuals. However, the relationships between post RYGB improvement in MASLD and multi-omics are unclear.

**Added value of this study**

To our knowledge, few studies assessed changes in liver histology and gene expression post-RYGB and their relationships with changes in metabolome and microbiome. Using a multi-omics approach, our study investigated these relationships in the context of MASLD improvement post-RYGB.

**Implications of all the available evidence**

The study results suggest that RYGB is associated with shifts in hepatic gene expression, serum/faecal metabolome, and intestinal microbiome that can be associated with improvements in liver histology, particularly biological pathways, such as those related to fatty acid metabolism, tricarboxylic acid cycle, and acylcarnitines. Our findings offer a framework for future investigations aimed at targeting these pathways to improve MASLD.

## Introduction

Obesity continues to rise globally and by 2050, over half of the global population is expected to be living with overweight or obesity.^1^ This increases the prevalence of obesity-related comorbidities such as metabolic dysfunction–associated steatotic liver disease (MASLD) and steatohepatitis (MASH),^2^, ^3^, ^4^ with MASH cirrhosis being the leading indication for liver transplantation.^5^ In addition to weight loss and exercise, pharmacological treatments targeting MASLD or obesity, such as resmetirom and glucagon-like peptide 1 receptor agonists, can be considered but need to be administered on a long-term basis, are costly and can be associated with side effects.^6^^,^^7^ Bariatric surgery, with Roux-en-Y gastric bypass (RYGB) being the most common procedure, is currently considered the best obesity treatment in those meeting the criteria.^8^ It has been shown to be effective for weight loss and improvement of metabolic parameters and obesity-related co-morbidities, including MASLD.^8^ Research has found that hepatic steatosis, hepatocellular ballooning, lobular inflammation, fibrosis, and non-alcoholic fatty liver disease score (NAS) improved significantly 12 months after surgery with remission rate of MASH over 85%, based on liver biopsy.^9^ However, the mechanism by which the bariatric surgery improves MASLD/MASH is poorly understood.

RYGB is a restrictive and malabsorptive procedure that alters nutrient flow, bile acid delivery, gut hormone secretion, faecal microbiome,^10^ metabolome^11^ and hepatic transcriptome^12^; all of which may play a role in MASLD pathogenesis and improvement post-RYGB.^13^ However, human studies using a multi-omic approach to investigate the effect of bariatric surgery on liver histology related to MASLD are very limited. We found only two studies assessing the relationships between hepatic genes and circulating metabolites but not with liver histology, metabolic parameters or faecal microbiome. One study in 31 patients with NASH (previous terminology for MASH) and 31 patients without NASH undergoing sleeve gastrectomy, found smaller mitochondria and reduced expression of proteins involved in oxidative phosphorylation in those with NASH compared to the control prior to the surgery.^14^ One-year post-surgery, NASH resolved in all patients. This was accompanied by restoration of mitochondrial function and oxidative phosphorylation, which was characterised by the increase in related protein levels and reduction or normalisation of metabolites such as α-ketoglutaric acid (an intermediate in tricarboxylic acid (TCA) cycle and glutaminolysis), branched chain amino acids (BCAAs) and other amino acids.^14^ In that study, authors used western-blot approach for targeted measurement of proteins pre- and post-surgery, but low analytical quality in some paired liver biopsies prevented them from transcriptomic assessment.^14^ Therefore, global and coordinated gene expression (at the RNA level) underlying these protein-level changes remains unclear. To our knowledge, the only other comparable study is by Lalloyer et al.,^12^ who assessed hepatic transcriptome and plasma metabolites related to cholesterol metabolism in a population of 26 patients (including 7 without steatosis and 14 with MASH) undergoing RYGB who were followed up for 12 months. They reported improvement in histology, coordinated changes in hepatic cholesterol and bile acid metabolism, and alteration in circulating cholesterol metabolites post-RYGB. This study did not assess the faecal metabolome or microbiome.^12^ To our knowledge, no published study has comprehensibly examined the changes in hepatic transcriptome, metabolome, and faecal microbiome as well as their interactions with changes in liver histology and metabolic parameters, within the same individuals following RYGB.

To address this gap, we performed a global transcriptome analysis to identify pivotal genes associated with RYGB-mediated improvement in liver histology. In addition, modules of co-expressed genes were identified among the differentially expressed genes (DEGs) and pathway enrichment analysis was performed to identify biological pathways potentially associated with these changes. These transcriptomic findings were further complemented by metabolome and microbiome data to uncover potential coordinated alterations across hepatic, metabolic and microbial pathways associated with RYGB-induced improvements in liver phenotype.

## Methods

### Study design

This cohort is part of a larger prospective cohort study of patients undergoing RYGB between 2013 and 2020 at University Health Network (UHN) in Canada who met the NIH criteria for RYGB.^15^ Patients with body mass index (BMI) ≥40 kg/m^2^ or BMI ≥35 kg/m^2^ with at least one comorbidity were included. All patients regardless of their sex who met the inclusion and exclusion criteria were approach for participation in this study. Inclusion criteria were body mass index (BMI) ≥40 kg/m^2^ or BMI ≥35 kg/m^2^ with at least one comorbidity, age >18 years, alcohol intake <20 g/d and on stable medication regimen for >3 months for hyperlipidaemia or type 2 diabetes prior to study entry. Only patients with complete data for liver histology, clinical parameters, faecal microbiome, serum/faecal metabolites, and hepatic transcriptome were included in the analysis. Exclusion criteria were liver disease other than MASLD; medications known to precipitate MASH 6 months prior to entry; regular intake of non-steroidal anti-inflammatory drugs; prebiotics, probiotics or antibiotics or any experimental drug in the 3 months prior to study entry; type 1 diabetes, chronic gastrointestinal diseases, previous gastrointestinal surgery modifying the anatomy (prior to bariatric surgery), smoking; pregnancy or breastfeeding.

### Ethics

Written informed consent was obtained from all participants. The study was conducted in accordance with the Declaration of Helsinki. This study (registered as NCT01856465 on ClinicalTrials.gov) was approved by the UHN Research Ethics Board (#13-6115). No animals were used in the conduct of this study.

### Demographic, anthropometric, biochemical measurements, and dietary intake

Sex was self-reported for the purposes of this study. It was asked from the participants at the time of their recruitment; gender was not assessed. Anthropometric measurements including, weight, height, BMI, waist circumference, and systolic and diastolic blood pressure, were collected pre- and 12 months post-RYGB. Fasting glucose, insulin, HbA1c, lipid profile, and liver enzymes were measured using standard protocols at the UHN Laboratory Medicine Program. The homoeostasis model of assessment for insulin resistance (HOMA-IR) was calculated using glucose (mmol/L) X insulin (mU/L/22.5).^16^ Dietary intake pre- and post-RYGB was also analysed as described, previously.^17^

### Liver biopsy

Liver biopsies were performed using wedge biopsy technique at the time of RYGB and ultrasound-guided needle biopsy at 12 months post-RYGB similar to a previous study.^12^^,^^18^ Each liver biopsy was divided into 2 sections; one section for histology which was preserved in formalin and then, embedded in paraffin. The second section was frozen in RNA later at −80 °C and used for liver transcriptomics.

#### Liver histology

A liver pathologist blinded to the study reviewed the biopsies and scored them using the Brunt system.^19^ Normal liver was defined as hepatic fat <5% and no other histologic abnormalities. MASLD was defined as any individuals with >5% steatosis and at least one of the five cardiometabolic risks factors which are: overweight/obesity, impaired glucose homoeostasis, hypertension, hypertriglyceridaemia and low HDL. MASH was defined based on the presence of hepatocyte ballooning.^20^ Liver fibrosis was also evaluated using the Brunt system and scored as F0 (no fibrosis), F1 (zone 3 perivenular/perisinusoidal/pericellular fibrosis), F2 (same as stage 1 with periportal fibrosis), F3 (septal/bridging fibrosis), and F4 (cirrhosis). NAFLD activity score (NAS) was calculated using steatosis grade, lobular inflammation and ballooning.^21^

#### Liver transcriptome

##### RNA extraction, library preparation and sequencing

Total RNA was extracted and sequenced by Novogene Inc., USA. Total RNA was extracted and sequenced using the Trizol and its quality was assessed with an Agilent bioanalyzer 2100. For samples with RIN ≥4, library of mRNAs was prepared and sequenced using Illumina platform and paired-end sequencing. Reads with adaptor, poly-N reads as well as low-quality reads were removed to obtain clean raw data which were then mapped to reference genome using Hisat2 version 2.0.5. and read count was obtained using featureCounts version 1.5.0-p3. These experiments were conducted by Novogene Inc., USA.

### Stool collection

Stool samples were collected and frozen for faecal microbiome and metabolome analyses within 72 h of patients’ preadmission and 12-month post RYGB, as previously described.^22^

### Faecal metagenomic sequencing and bioinformatic analysis

Faecal DNA was extracted using the ZymoBIOMICS DNA Miniprep Kit (ZYMO RESEARCH, Irvine, CA, USA).^22^ DNA sequencing and bioinformatics analysis were performed by the Centre of the Analysis of Genome Evolution and Function (University of Toronto, ON, Canada) as previously described.^22^

### Metabolomic measurements

Faecal and serum metabolites were extracted and measured using a modified combination of direct injection mass spectrometry and a reverse-phase LC-MS/MS (The Metabolomic Innovation Centre, University of Alberta, AB, Canada).^22^ Serum metabolite concentrations were reported in μM. For faecal metabolites only, sample water content was determined by lyophilisation of one aliquot, followed by weighing before and after the drying process. Faecal metabolites were reported in μmol/g and were normalised with respect to the water content.

### Statistics

This study was part of a larger prospective study investigating the relationship between the intestinal microbiome and MASLD in patients undergoing RYGB. For this longitudinal analysis, patients were included if they returned for the follow-up liver biopsy which was performed at 12 months post-RYGB and had hepatic transcriptome data available at both baseline and 12 months. Statistical analyses were performed in R version 4.4.2. Differences in anthropometric and biochemical variables as well as dietary intake between pre- and 12-months post-RYGB were assessed using paired Wilcoxon signed-rank and McNemar, McNemar-Bowesky or Stuart–Maxwell Marginal Homogeneity test as appropriate in gtsummary version 2.3.0. p value < 0.05 was considered significant.

#### Transcriptome data quality control

To evaluate distributional differences between pre- and post-RYGB transcriptome samples, we generated log fold-change versus mean expression plot and density plot at both raw and normalised stages. Raw read counts obtained from RNASeq were first transformed to logCPM using the edgeR and limma packages in R. Log-intensity ratios versus average expression (MA) plots were created to visualise potential systematic biases between groups. Density plots of logCPM distributions across all samples were generated to assess library size effects and compositional differences.

#### DEG analysis

Genes were prefiltered to 16,504 genes to include only those which had at least 9 non-zero samples in at least one timepoint. Paired analysis of differential gene expression was performed using edgeR version 4.4.2 and limma version 3.62.2 with default settings. Read counts were first transformed using voom. Then, limma was used for linear modelling with empirical Bayes moderation. To account for repeated measures within the same subjects pre- and post-RYGB, we used duplicateCorrelation to estimate within-subject correlation and applied the limma voom transformation. Linear modelling was then performed with lmFit and eBayes, incorporating the estimated correlation. Residual diagnostics with plotSA showed no systematic trend, supporting model assumption of homoscedasticity. p-values were adjusted using Benjamini-Hochberg method to account for multiple hypothesis testing and log2FoldChange was calculated. DEG were identified using p-adjusted<0.05 and |log2FC|≥1.

#### Transcriptome functional enrichment

Gene set enrichment analysis with Kyoto Encyclopedia of Genes and Genomes database (GSEA KEGG) was performed using ClusterProfiler version 4.14.6. KEGG pathways with |normalised enrichment score (NES)|>1 and q < 0.05 were considered significant. Top 30 pathways based on the q value were visualised using ggplot2 version 3.5.2.

#### Weighted Gene Co-expression Network Analysis (WGCNA)

WGCNA (version 1.73) was performed on all DEGs to identify significant up- or downregulated modules. The soft-thresholding power was selected using pickSoftThreshold. A signed network was constructed using β = 7, based on the model fit curve with R^2^ = 0.70 used as the reference criterion. Log2FoldChange was calculated and q < 0.05 was used to determine significance. To focus our analysis on WGCNA modules specifically associated with RYGB, gene significance was calculated for each gene as the −log_10_-transformed p-value derived from the linear regression between normalised gene expression values and pre/post-RYGB status. Module significance (MS) was then calculated as the average gene significance across all genes within each module. The five modules with the highest module significance values were selected for downstream analyses.

#### Hallmark pathway analysis

Hallmark analysis was performed on the top 5 modules with the highest MS value using MSigDBR database. Hallmarks with |NES|>1 and q < 0.05 were considered significant. Gene set variation analysis (GSVA) was performed on the significant hallmarks and change in each hallmark pathway was correlated with change in NAS using Spearman Correlation coefficient in rstatix package version 0.7.2.

#### Microbiome analysis

MaAsLin 3 (version 0.99.9 was used to identify species whose abundances significantly differed between timepoint. Models for MaAsLin 3 included timepoint as a fixed effect and subject as a random effect for abundance models. Otherwise, default parameters were used for Maaslin3. Data were visualised using the ggplot2 (version 3.5.1) and ggforce (version 0.4.2) packages in R Studio version 2024.12.0 + 467 running R version 4.4.2.

#### Metabolites

Faecal and serum acylcarnitines and intermediate metabolites involved in fatty acid degradation, tricarboxylic acid cycle and valine, leucine and isoleucine degradation were compared between groups using paired Wilcoxon signed-rank test in gtsummary package version 2.3.0 and adjusted using Benjamini-Hochberg method. q < 0.05 was considered significant.

#### Correlations

To correlate expression levels of the genes with other variables, normalisation of the transcriptome data is required. Raw transcriptome counts were normalised using fragments per kilobase of transcript per million mapped reads (FPKM) which adjusts for both gene length and sequencing depth. Associations between change in DEG, bacteria, metabolites, histology, dietary intake, and clinical parameters were assessed using Spearman Correlation coefficient using rstatix package version 0.7.2. All data were analysed using R version 4.4.2. Correlations were adjusted using Benjamini-Hochberg method and both raw and adjusted p value were reported. In a sensitivity analysis, these correlations were also repeated in female patients using similar approach.

#### Regression

For key identified associations, we performed regression using lm function with backward elimination and accounted for covariates percent excess BMI loss (%EBMIL), HbA1c, and baseline fibrosis as well as the baseline values of the predictor and outcome. %EBMIL was calculated using:

$$\%EBMIL=\frac{BMI\;at\;baseline-BMI\;at\;12\;month\;}{BMI\;at\;baseline-25\;}\times 100$$

When covariates were highly correlated with each other, the most clinically appropriate covariate was retained to reduce multicollinearity. Full models were first fitted with the selected covariates. Backward elimination was then performed by sequentially removing the covariate with the highest p-value. Model reduction was continued only when removal of the covariate decreased the Akaike information criterion (AIC). If AIC increased after removal, the previous model with the lowest AIC was retained as the final model.

#### Network analysis

Network analysis was performed using Cytoscape (v3.10.1). Hub nodes were identified using the CytoHubba plugin based on Maximal Clique Centrality.

#### Stratified analysis by weight loss

For each patient, %EBMIL was calculated.^23^ Next, patients were divided into two groups based on 60% EBMIL cutoff.^23^ Change in normalised count (in fpkm) of the genes of interest was calculated and compared between the two groups using Wilcoxon rank-sum tests. P-values were adjusted for multiple testing using the Benjamini-Hochberg method.

#### Multi-block analysis

We used DIABLO from the mixOmics R package (version 6.34.0) to assess whether coordinated changes in hepatic transcriptome, metabolites, and intestinal microbiome features distinguished participants with resolved NAS (either continued to have NAS of 0 or their NAS was reduced to 0 post-RYGB) from those with persistent NAS (NAS>1 post-RYGB). Raw transcriptome counts were normalised using edgeR (version 4.8.2). Change scores of the transcriptome, metabolome, and microbiome were calculated. Due to small sample size and to avoid excluding patients who did not have serum data, only faecal metabolome was analysed. Variables with missing values or zero variance were removed. Prior to DIABLO integration, we examined correlation between omics blocks using pairwise partial least-squares analyses. For each pairwise comparison, 25 features were retained from each omics block. Next, the gene block (with 16,504 genes) was restricted to the genes from the module of interest before model tuning. NAS resolution status was used as the supervised outcome. Pairwise partial least squares (PLS) analyses were first used to assess cross-block associations, followed by DIABLO using a conservative design matrix with off-diagonal values of 0.1. Model components and selected features were tuned using repeated 5-fold cross-validation and repeated 10 times.

### Role of funders

The funders (Canadian Institutes of Health Research (MOP-126139) and American College of Gastroenterology Bridge Award) had no role in any aspects of the study including study design, data collection, analysis, interpretation of the findings, writing the manuscript or decision or publication of this paper.

## Results

### RYGB induced weight loss and improved metabolic profile

Thirty-eight adults with obesity (92% female; median age 50 yr) were included in this analysis and completed paired assessments before and 12 months after RYGB. BMI, weight, waist circumference, blood pressure, ALT (but not AST or ALP), glycaemic control measurements and fasting triglycerides, HDL, and LDL cholesterol significantly improved 12-months post-RYGB. Liver histology mirrored the clinical benefit: median steatosis decreased from 22.5% to 0% (paired Wilcoxon signed-rank test*,* p < 0.001), hepatocyte ballooning resolved in 35/38 participants, and the proportion of patients with NAS ≥5 fell from 16% to 0%. This data is summarised in Supplementary Table S1. Fig. 1 shows representative pre- and post-RYGB liver histology images demonstrating histological resolution compared to persistent histological abnormalities.

**Fig. 1:**
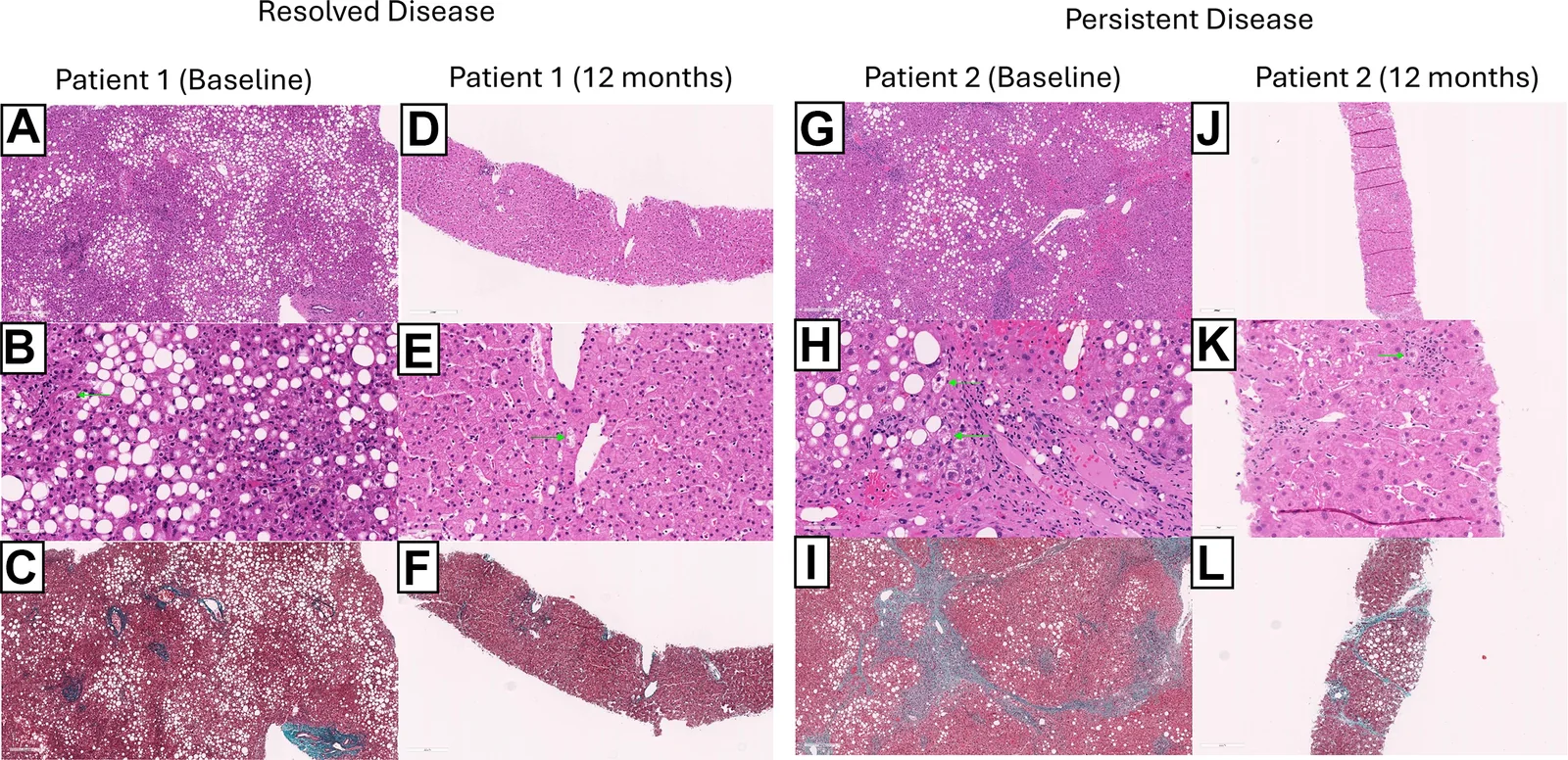
Representative histology images of a patient with resolved disease versus persistent disease. Patient 1 (Baseline): A, B) HE stain: shows moderate to severe macrovesicular steatosis (A), and hepatocyte ballooning (B, arrows). C) Masson trichrome stain highlights perivenular and periportal fibrosis (stage 2/4, Kleiner and Brunt). Patient 1 (12 months): D, E) HE stain: Large fat droplets present in less than 5% of hepatocytes (D, E), and rare hepatocyte ballooning (E, arrow). F) Masson trichrome stain highlights perivenular fibrosis only (stage 1/4, Kleiner and Brunt). Patient 2 (Baseline): G, H) HE stain: shows moderate macrovesicular steatosis (G), and frequent hepatocyte ballooning (H, arrows). I) Masson trichrome stain highlights severe bridging fibrosis with complete nodules (stage 4/4, Kleiner and Brunt). Patient 2 (12 months): J, K) HE stain: Large fat droplets present in less than 5% of hepatocytes (J, K), and hepatocyte ballooning (K, arrow). L) Masson trichrome stain highlights bridging fibrosis with few round septa (incomplete nodules), stage 3–4/4 (Kleiner and Brunt).

### RYGB was associated with changes in hepatic transcriptome

To evaluate the effects of RYGB on hepatic gene expression, we performed RNA sequencing (RNASeq) on paired liver biopsies obtained at baseline and 12 months post-surgery. First, we performed quality control analysis on the hepatic transcriptome data at pre- and post-RYGB with and without normalisation. Prior to normalisation, log fold-change versus mean expression plot and density plot revealed potential asymmetry in distributional differences pre- and post-RYGB samples (See Supplementary Figure S1A and C). However, after normalisation, log fold-change versus mean expression plot centred around zero and density distributions between groups overlapped suggesting that any potential differences due to unequal library sizes and compositional effects were further minimised (Supplementary Figure S1B, D and E), and therefore, there was no evidence of a systematic upward shift in post-RYGB samples. Thus, the data was suitable for the downstream differential expression analysis. Finally, the results are presented in the context of methodological differences in pre- and post-RYGB liver biopsies which could potentially influence transcriptomic profile.^24^ This could not be modelled as an independent co-variate because of the confounding effect of time post-RYGB. Given this methodological difference, the transcriptomic results should be interpreted cautiously.

Paired differential expression analysis identified 13,353 significantly differentially expressed genes (DEGs) (,|log_2_FoldChange| ≥ 1, FDR <0.05) between baseline and 12 months post-RYGB (Fig. 2A and Supplementary Excel Sheet DEG). Among DEGs, the genes with the largest positive log_2_FoldChanges were ALB, MT–CO1 (mitochondrial), MT–ND4 (mitochondrial), MT-RNR2 (mitochondrial), SERPINA1, HP, FGA, C3, MT-CYB (mitochondrial), and MT–CO3 (mitochondrial). Several of these genes, including MT–CO1, MT–CO3, MT-CYB, and MT–ND4, encode components of the respiratory chain complexes (i.e. electron transport chain) in the mitochondria and contribute to energy production. The genes with the largest negative log2 fold-changes were LPL, EGR2, TREM2, SIK1, VGLL3, BOC, MS4A2, AL358232.1, CHRDL1, and SHISA3. To contextualise the global transcriptomic shifts post-RYGB, GSEA using the KEGG database identified 166 significantly enriched pathways (Supplementary Excel Sheet GSEA KEGG). Among the most prominent were “complement and coagulation cascades”, “fatty acid degradation”, and “valine, leucine and isoleucine degradation” (Fig. 2B).

**Fig. 2:**
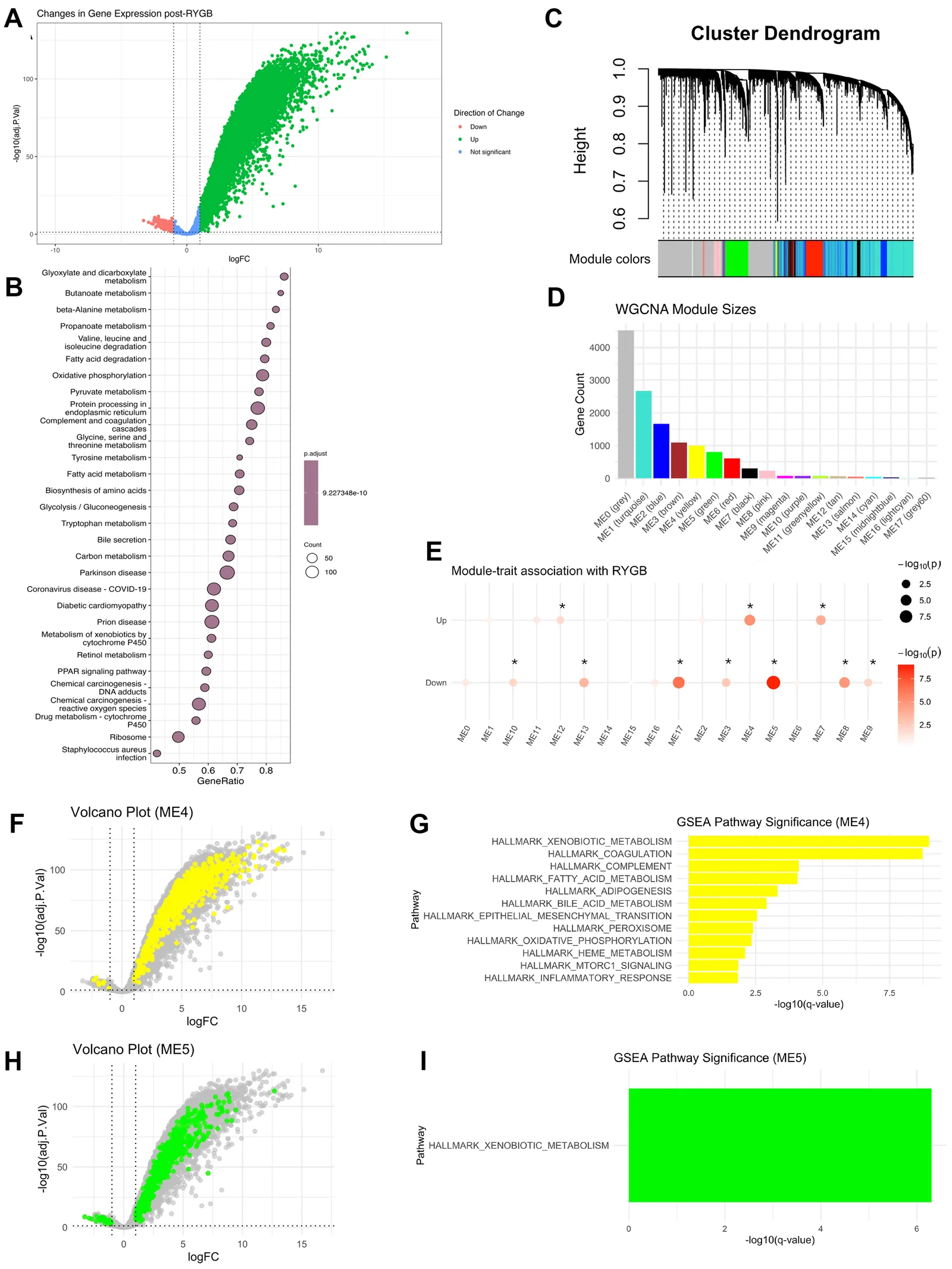
Transcriptome signature 12 months post-RYGB compared to baseline (n = 38 patients). A) Volcano plot of DEGs with |logFC|≥1 and adjusted p < 0.05 [limma moderated t-test; Benjamini–Hochberg adjusted]. B) Top 30 GSEA KEGG pathways. C) WGCNA cluster dendrogram performed on 13,353 DEGs. D) Size of each WGCNA module (i.e. number of genes in each module). E) Direction of change in each module post-RYGB (Spearman Correlation). F) Volcano plot of ME4 DEGs shown in yellow and other genes in grey. G) Hallmark pathway analysis of ME4. H) Volcano plot of ME5 DEGs shown in green and other genes in grey. I) Hallmark pathway analysis of ME5. ∗ showed adjusted p < 0.05.

Next, to explore coordinated RYGB-associated gene expression changes, we applied WGCNA, which clustered genes into modules based on co-expression patterns. Overall, 7 modules were downregulated and 3 were upregulated following RYGB (Supplementary Excel File WGCNA MEs). The most upregulated module was ME4 (limma moderated t-test, log2FoldChange = 0.11 and adjusted p < 0.0001), and the most downregulated module was ME5 (limma moderated t-test, log2FoldChange = −0.14 and adjusted p < 0.0001). To further examine the relationship between gene co-expression modules and the surgical intervention, we conducted a module significance analysis. Among all modules, ME17, ME5, ME8, ME7, and ME4 exhibited the highest module significance values of 3.89, 3.82, 2.71, 2.54, and 2.51, respectively, indicating the strongest association with the impact of surgery (Supplementary Excel File ModuleSignificance). Next, we performed hallmark pathway enrichment analysis on the top 5 modules to reveal biologically distinct patterns in specific co-expressed modules. No hallmark pathways were identified in ME17 and ME7. Myogenesis pathway was identified in ME8, but no metabolism-related pathway was enriched in this module (Supplementary Excel File ME8). ME4 and ME5 were associated with RYGB (Fig. 2C–E). The genes involved in each module (ME4 and ME5) were transposed onto a volcano plot (Fig. 2F and H) and the hallmark pathway analysis was also performed on ME4 and ME5 (Fig. 2G and I). In ME4, several hallmark pathways were found to be significant which can be broken into 5 main processes.^25^ The first group is metabolic process, which includes xenobiotic metabolism, fatty acid metabolism, bile acid metabolism, oxidative phosphorylation and haem metabolism. The second is immune process and includes coagulation, complement and inflammatory response pathways. The third group is signalling process which includes epithelial mesenchymal transition and MTORC1 signalling. We also found adipogenesis (development process) and peroxisome (cellular component process) in ME4 (Fig. 2G). In ME5, only one pathway, xenobiotic metabolism, was identified as significant (Fig. 2I).

Some of the above pathways such as those in metabolic and immune processes are implicated in MASLD and obesity. To determine which hallmark pathway was most closely associated with MASLD-related histological improvement post-RYGB, we correlated the hallmark pathways with changes in NAS score (Supplementary Table S2). Among pathways identified in ME4 and ME5, we found three pathways of coagulation, adipogenesis, and fatty acid metabolism correlated significantly with change in NAS. However, the change in fatty acid metabolism hallmark pathway showed the strongest association with change in NAS post-RYGB (Spearman's ρ = 0.38, p = 0.019). The covariate-adjusted regression analysis showed a significant positive association between this pathway and change in NAS (linear regression model, β = 1.44, SE = 0.69, p = 0.0499, covariates: % EBMIL and change in HbA1c). Therefore, we focused on this pathway for further analyses.

### Specific hepatic transcriptomic signatures related to fatty acid metabolism were associated with specific changes in liver histology and metabolites post-RYGB

Ten hepatic genes were identified as core enrichment genes in fatty acid metabolism hallmark pathway. These genes were involved in 1) fatty-acid β-oxidation/branch chain amino acid (BCAA) degradation (ACADVL, ACOX1, EHHADH, and ALDH3A2); 2) TCA cycle (SUCLG2, SDHC); 3) retinoid and aldehyde metabolism in detoxification and lipid signalling (ALDH1A1, ALDH9A1, and RETSAT); and 4) membrane-lipid biosynthesis (SERINC1) which modulates phospholipid composition. Their role in fatty acid degradation, BCAA degradation and TCA cycle are shown in Fig. 3. It is important to note that many of these 10 core enrichment genes identified in fatty acid metabolism hallmark pathway were also identified in other hallmark pathways related to metabolism and immune processes.

**Fig. 3:**
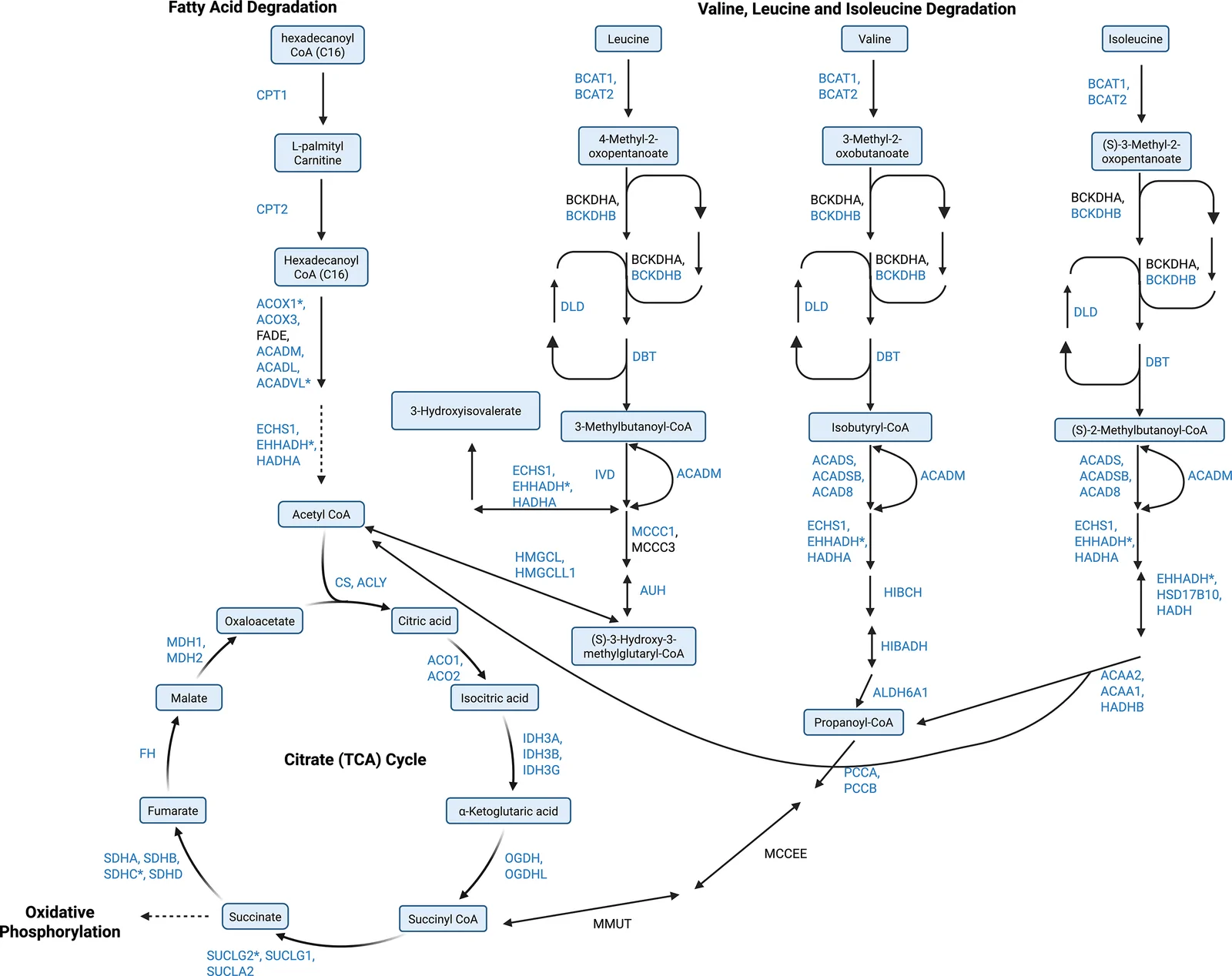
Schematics of pathways involved in BCAA degradation, fatty acid metabolism and citrate cycle (n = 38 patients). Enzymes identified as DEG are shown in blue. Fatty acid metabolism core enrichment genes are shown with ∗. Created in BioRender. Ghorbani, Y. (2026) https://BioRender.com/s1i55do.

We then investigated if changes in these 10 hepatic genes were related to changes in liver histology post-RYGB. Overall, we found that upregulation in β-oxidation and BCAA degradation genes (*ACADVL, EHHADH, ACOX1*) were significantly positively correlated with less histological improvement, especially inflammatory injury, whereas TCA cycle genes (*SUCLG2, SDHC*) and the membrane-lipid transporter *SERINC1* were correlated negatively to the greater resolution of hepatocyte ballooning (see Fig. 4 and Supplementary Excel File DEGandHistology). However, only correlation between change in ACADVL or ALDH9A1 and improvement in ballooning remained significant after adjustment for multiple hypothesis testing (Spearman correlation, adjusted p = 0.0471 for both) while correlation between SDHC or SERINC1 and ballooning had a trend (Spearman correlation, adjusted p = 0.0701 for both). In a sensitivity analysis, we assessed the correlation between these genes and histological features in females (n = 35) and found that changes in several of the genes were also correlated with change in ballooning (Supplementary Excel File DEGandHistologyFemales). We were not able to perform these correlations in males due to their small group (n = 3).

**Fig. 4:**
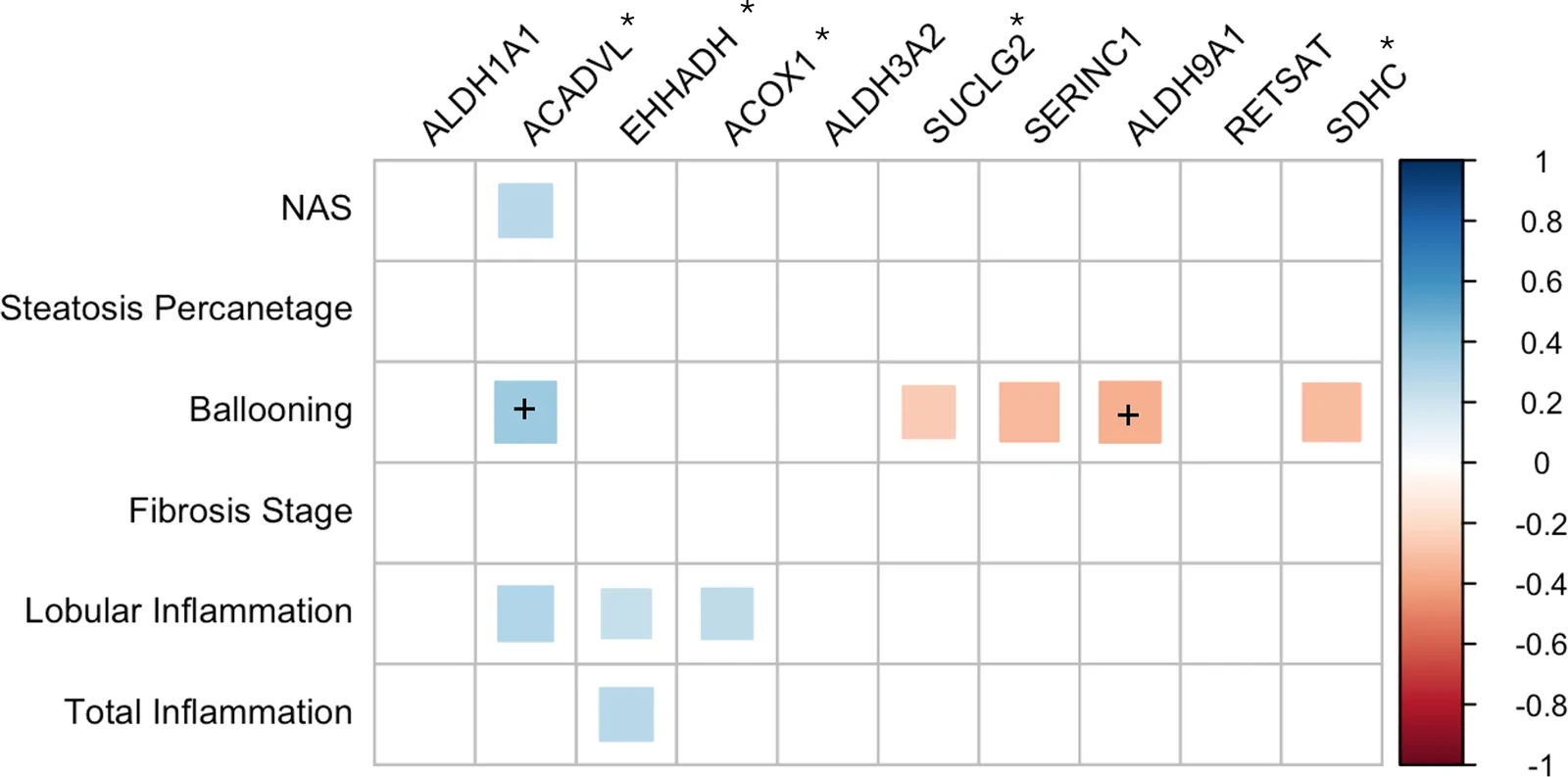
Correlation between change in DEGs and change in histological features (n = 38 patients). ∗ denotes core enrichment genes in fatty acid metabolism hallmark. Only significant correlations are shown (Spearman Correlation, unadjusted p < 0.05). + denotes adjusted p < 0.05 after adjustment for multiple hypothesis testing using Benjamini-Hochberg method.

Considering that these 10 genes are potentially involved in fatty acid metabolism including fatty acid degradation, TCA cycle and BCAA-degradation, we focused our faecal and serum metabolomic data analysis on the intermediates involved in these processes and acylcarnitines which transport the fatty acids for downstream metabolism in the mitochondria. Targeted metabolomics showed broad declines in serum medium- and long-chain acylcarnitines and TCA intermediates post-RYGB (see Supplementary Tables S3 and S4). For example, C5 fell from 0.142 μmol L^−1^ to 0.087 μmol L^−1^ (paired Wilcoxon signed-rank test, q = 0.0001) and C10:2 from 0.077 to 0.054 μmol L^−1^ (paired Wilcoxon signed-rank test, q = 0.0001). Conversely, the short-chain product C4:1 rose (0.054–0.074 μmol L^−1^; paired Wilcoxon signed-rank test, q = 0.0047). In contrast to serum, 29 of 38 quantified faecal acyl-carnitines increased after RYGB, for example, C8 rose three-fold (1.18 × 10^−4^–3.54 × 10^−4^ μmol g^−1^; paired Wilcoxon signed-rank test, q < 0.0001) and C5DC more than tripled to 3.88 × 10^−4^ μmol g^−1^ (paired Wilcoxon signed-rank test, q < 0.0001). Among faecal TCA- and BCAA-related metabolites, only faecal citric acid remained significantly decreased after multiple-comparison adjustment (from pre-RYGB 123 μmol g^−1^ to post-RYGB 112 μmol g^−1^; paired Wilcoxon signed-rank test, q = 0.0116) whereas changes in other TCA intermediates and BCAAs did not reach statistical significance. Among faecal TCA-cycle intermediates, faecal citric acid was markedly reduced from 123 μmol g^−1^ to 112 μmol g^−1^ (paired Wilcoxon signed-rank test, q = 0.0116) whereas shifts in other TCA intermediates and BCAA did not reach statistical significance after multiple-comparison adjustment (Supplementary Tables S5 and S6).

To demonstrate that the changes in these acylcarnitines and metabolites were not due to alterations in dietary intake post-RYGB, we investigated whether there were potential associations with energy and macronutrient intakes from collected 3-day food records pre- and 12 months post-RYGB. The carbohydrate intake significantly decreased from 207 g per day to 124 g per day at 12 months post-RYGB (paired Wilcoxon signed-rank test, p = 0.014) (Supplementary Table S7). Although there were some correlations between carbohydrate intake and the acylcarnitines, these associations were limited and did not remain significant after adjustment (See Supplementary Excel File DietCorrelation). Within the limitations of the self-reported dietary intake (e.g. underreporting or misreporting), it is unlikely that change in dietary intake affected serum and faecal acylcarnitines and metabolites. However, due to the nature of the RYGB surgery, which is a restrictive and malabsorptive procedure, it is possible that the observed increase in faecal acylcarnitines was influenced by the malabsorption of dietary carnitines.

We then investigated whether changes in the serum and faecal acylcarnitines as well as TCA- and BCAA-related metabolites were associated with changes in the ten enrichment genes and liver histology post-RYGB. Correlation heat maps (Fig. 5 A-B) demonstrated that more increase in β-oxidation gene expression was significantly associated with smaller reductions in serum medium-/long-chain acyl-carnitines. However, the correlation between the genes and serum metabolites did not remain significant after adjustment (Supplementary Excel File DEGandSerumMetabolites) while among faecal metabolites, only faecal C18:1OH remained significantly correlated with SDHC (Spearman's ρ = 0.50, adjusted p = 0.0015; See Supplementary Excel File DEGandFaecalMetabolites). Some of these correlations remained significant in females (Supplementary Excel File DEGandSerumMetabolitesFemales and DEGandFaecalMetabolitesFemales) but not after adjustment for multiple hypothesis testing. Additionally, changes in specific metabolites (Fig. 6A–B) were associated with histological improvements; for example, a decrease in serum short and medium-chain acyl-carnitines was associated with a reduction in hepatic ballooning post-RYGB and there was a trend towards significance after adjustment (Supplementary Excel File HistologyandSerumMetabolites). Conversely, an increase in faecal acyl-carnitines was associated with improvement in hepatic ballooning (Supplementary Excel File HistologyandFaecalMetabolites). In regression analyses of the most significant serum metabolite–histology associations, the following metabolites were significantly associated with ballooning: 1) reduction in serum C6:1 (linear regression model, covariates: %EBMIL, change in HbA1c and baseline fibrosis stage; β = 36.04, SE = 16.20, p = 0.039); and 2) reduction in faecal C3:1 (linear regression model, covariates: %EBMIL, change in HbA1c and baseline fibrosis stage; β = −1896, SE = 781, p = 0.0219).

**Fig. 5:**
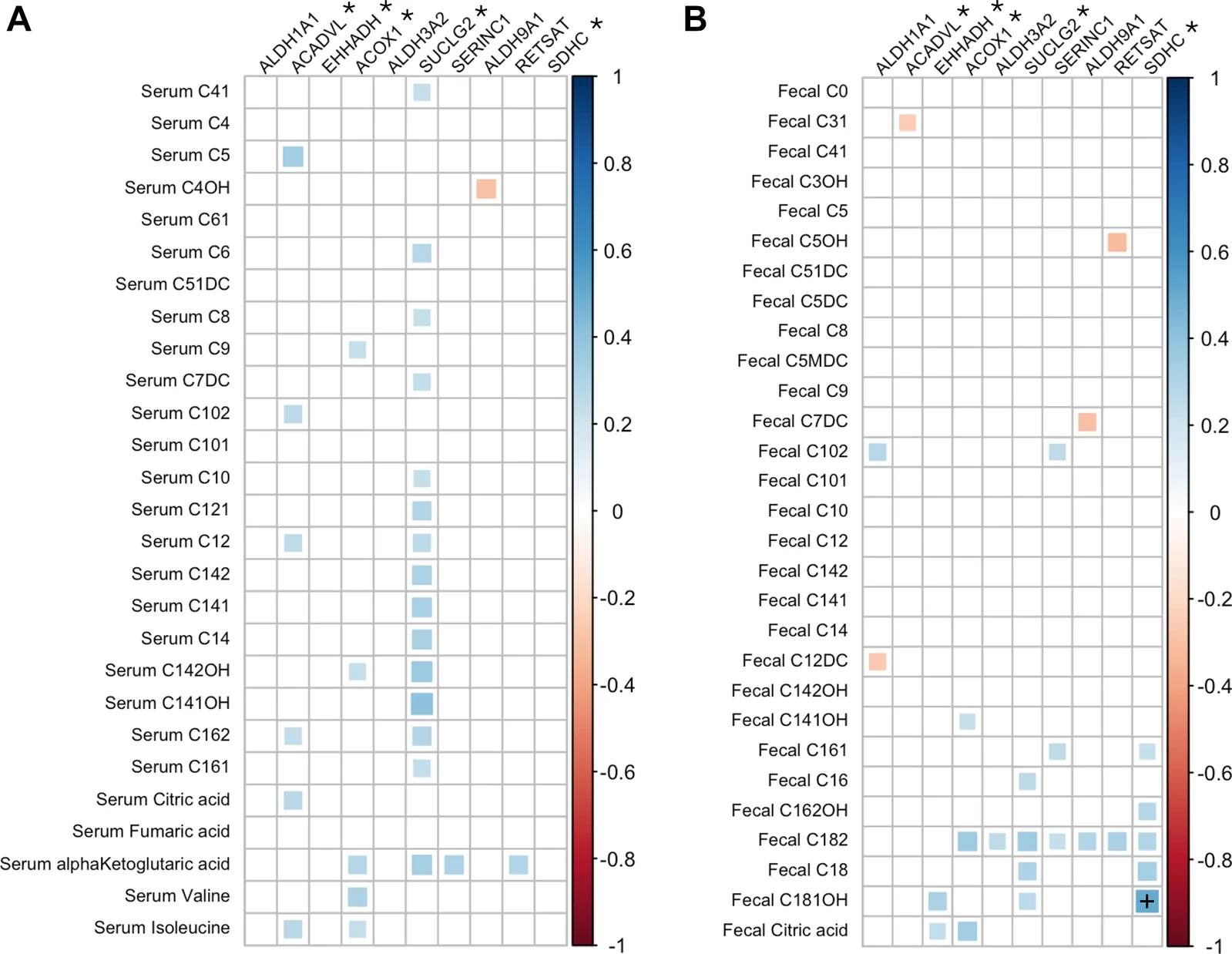
Heat map of correlations between changes in enrichment genes and A) changes in significant serum acylcarnitines and TCA metabolites and B) changes in significant faecal acylcarnitines and TCA metabolites (n = 38 patients). ∗ denotes core enrichment genes in Fatty acid metabolism hallmark. Only significant correlations are shown (Spearman Correlation, unadjusted p < 0.05). + denotes adjusted p < 0.05 after adjustment for multiple hypothesis testing using Benjamini-Hochberg method.

**Fig. 6:**
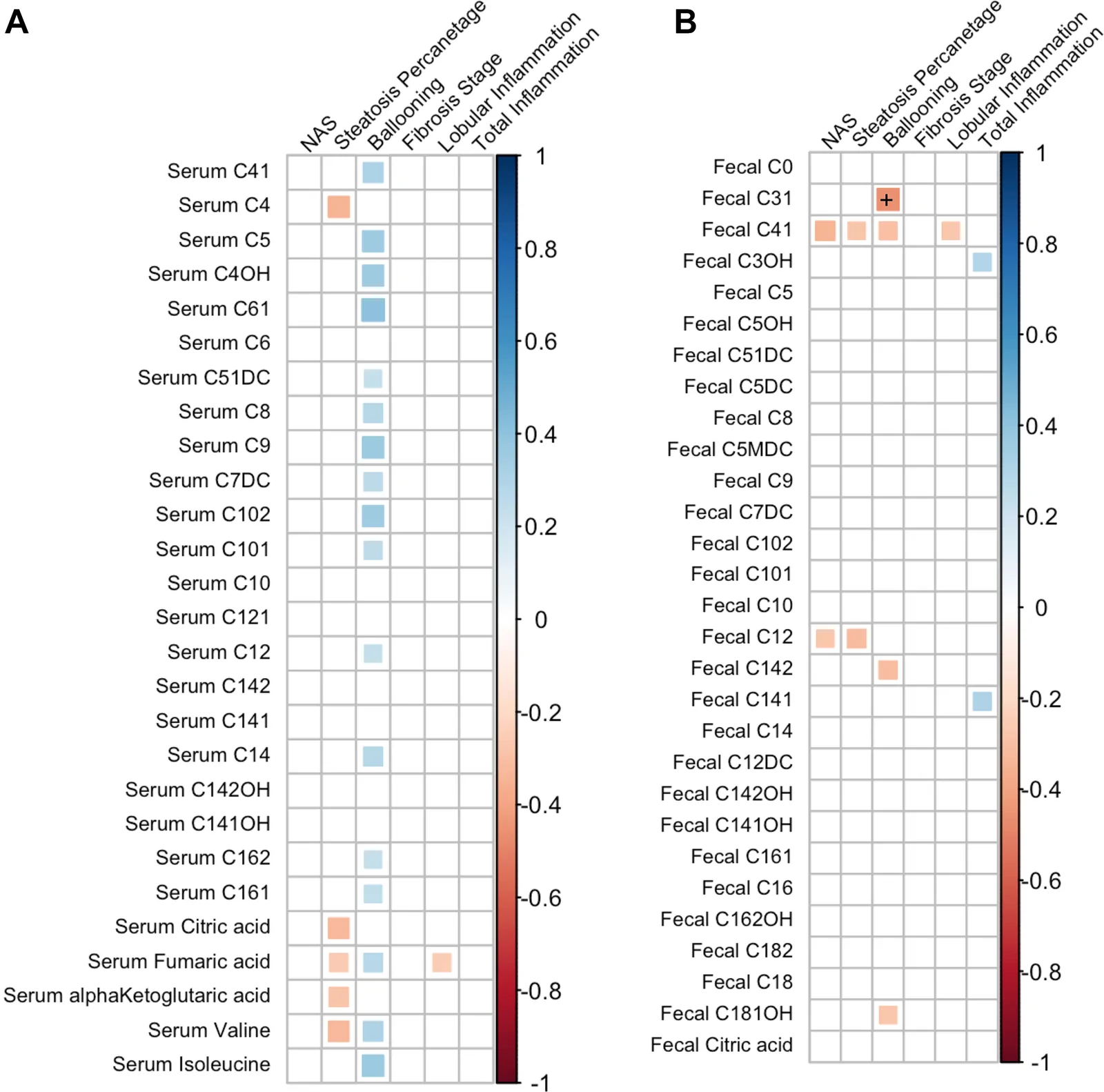
Heat map of correlations between change in hepatic improvement and A) significant changes in serum acylcarnitines and TCA metabolites and B) significant changes in faecal acylcarnitines and TCA metabolites (n = 38 patients). Only significant correlations are shown (Spearman Correlation, unadjusted p < 0.05). + denotes adjusted p < 0.05 after adjustment for multiple hypothesis testing using Benjamini-Hochberg method.

Finally, the global transcriptome analysis showed upregulation in several other genes involved in fatty acid degradation, BCAA degradation and TCA cycle. These genes also correlated significantly with liver histology in addition to change in faecal and serum acylcarnitines and BCAA and TCA-related metabolites (Supplementary Excel File OtherGeneAndHistology and Supplementary Excel File OtherGeneAndMetabolites) but did not remain significant after adjustment.

### Intestinal microbiome shifts were associated with changes in acylcarnitines

First, we looked at the overall changes in bacterial composition post-RYGB. Shotgun metagenomics found depletions in butyrate producers *Anaerostipes hadrus* (−1.93 log-fold; mixed-effects model, q < 0.0001) and *Bifidobacterium longum* (−1.91 log-fold; mixed-effects model, q = 0.015) post-RYGB (see Supplementary Table S8). It also found enrichments in, specifically, *Roseburia hominis* (+1.53 log-fold; mixed-effects model, q = 0.020) as well as *Alistipes putredinis*, *Escherichia coli, Streptococcus parasanguinis,* and *Streptococcus salivarius* (see Supplementary Table S8 and Fig. 7 A-E). Some of these bacteria are capable of using faecal acyl-carnitines and TCA intermediates like citric acid as a substrate.^26^^,^^27^ Thus, we wanted to investigate whether changes in faecal levels of these metabolites correlated with specific changes in the relative abundance of these bacterial taxa post-RYGB. Abundances of all 5 post-RYGB-enriched bacteria correlated significantly with some of the faecal metabolites; notably, we found that increases in *R. hominis* abundance, a species capable of β-oxidising carnitines, as well as *S. parasanguinis,* correlated with rises in multiple faecal acyl-carnitines (Fig. 7F and Supplementary Excel File IMandFaecalMetabolites). Some of these correlations remained significant in the female population (Supplementary Excel File IMandFaecalMetabolitesFemales). Also, changes in abundance of these bacteria correlated significantly or with a trend, with the genes of interest and with histological features, but they did not remain significant after adjustment (Supplementary Excel IMandGenes and IMandHistology).

**Fig. 7:**
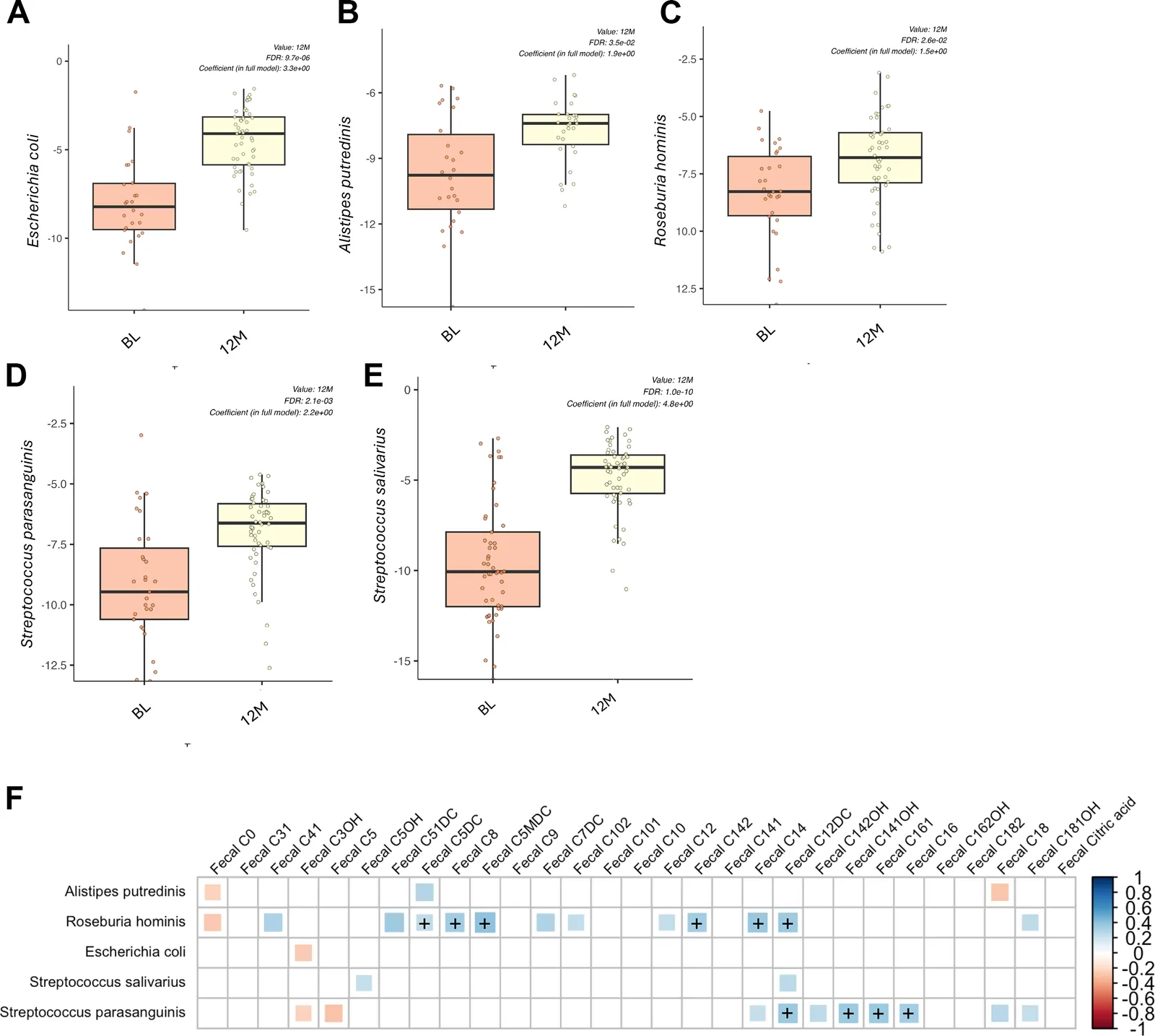
Microbiome shifts associated with acylcarnitines and TCA intermediates A–E) Change in bacteria potentially capable of using acylcarnitines/citric acid as substrate; F) Correlation between change in acylcarnitine/citric acid-using bacteria and change in faecal acylcarnitines and citric acid (n = 38 patients). Only significant correlations are shown (Spearman Correlation, unadjusted p < 0.05). + denotes adjusted p < 0.05 after adjustment for multiple hypothesis testing using Benjamini-Hochberg method.

### Multi-omic analyses identified associations among the hepatic transcriptome, metabolome, microbiome, and liver histology

Next, to examine the multi-omic relationship between liver histological features, metabolites, genes of interest and key bacteria, we constructed an integrated correlation network (Fig. 8). Hepatocyte ballooning was a highly connected histological feature in this network, and its improvement correlated with RYGB-related changes in expression of multiple mitochondrial and peroxisomal β-oxidation genes, including SUCLG2, SDHC, SERINC1, ALDH9A1, and ACADVL. These genes were connected to distinct groups of serum and faecal acylcarnitines, branched-chain amino acids and TCA cycle intermediates which were altered post-RYGB. Among the genes, SUCLG2 showed the greatest connectivity, linking to medium- and long-chain acylcarnitines as well as α-ketoglutaric acid. However, in regression analysis, there was no relationship between changes in SUCLG2 and changes in ballooning (linear regression model, β = −0.006, SE = 0.006, p = 0.318) when using as covariates, baseline fibrosis stage and change in HbA1c. For the microbiome, *R. hominis* and *S. parasanguinis* were linked to multiple faecal acylcarnitines (Fig. 7F and Supplementary Excel File IMandFaecalMetabolites) but several microbiome-gene and microbiome–histology associations did not remain significant after correction for multiple hypothesis testing.

**Fig. 8:**
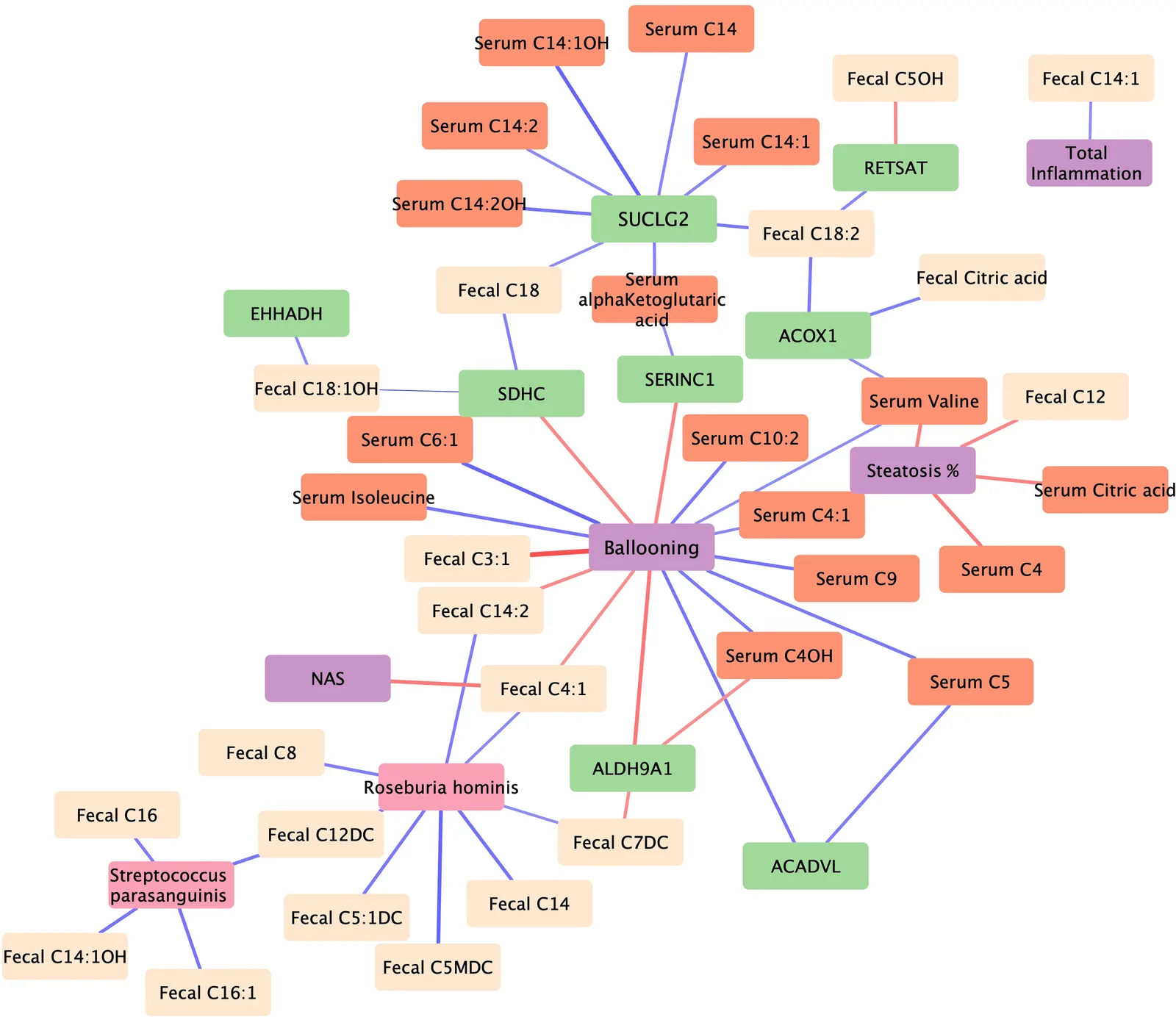
Network analysis of core enrichment genes in relation with faecal and serum metabolites, histology and key bacteria (n = 38 patients). Only interactions with p < 0.01 (Spearman Correlation) are shown. Blue lines indicate positive correlation and red indicates negative correlation. Genes are shown in green, serum metabolites in orange, faecal metabolites in light orange, histology in purple and bacteria in pink. The thickness of the line shows the strength of the correlation.

We also performed integrated multi-omic analysis using DIABLO to assess whether coordinated changes in hepatic transcriptome, metabolites, and intestinal microbiome features distinguished participants with resolved NAS (either continued to have NAS of 0 or their NAS was reduced to 0 post-RYGB) from those with persistent NAS (NAS >1 post-RYGB). Pairwise partial least-squares analyses showed strong correlations among changes in hepatic transcriptome, faecal metabolite, and microbiome features (see Supplementary Table S9 and Supplementary Figure S2). Using the hepatic gene block restricted to ME4 module (999 genes) as well as 47 faecal metabolites and 225 bacteria, the analyses identified partial separation between participants with resolved versus persistent NAS, with the clearest separation observed in the hepatic transcriptome block (Supplementary Figure S3). The final model selected features across the four components. These features included genes related to fatty acid remodelling (e.g. desaturation), insulin signalling and glucose transport, cellular signalling, and regulation of metabolism and mitochondrial function. It also selected features related to TCA cycle intermediates, BCAAs, and acylcarnitines, as well as multiple species related to *Streptococcus*, *Alistipes*, and *Bacteroides* (Supplementary Figure S4).

### Changes in the 10 core enrichment genes were not different between those with low versus high weight loss

To evaluate whether post-RYGB transcriptomic changes differed based on the magnitude of weight loss, participants were stratified into low (<60% EBMIL) and high (>60% EBMIL) weight loss groups. There were no significant differences in the change in expression of the 10 core enrichment genes between the two groups (Wilcoxon rank-sum test, p > 0.05) (Supplementary Table S10).

## Discussion

Obesity is often associated with several co-morbidities, including MASLD and multiple factors and mechanisms contribute to their pathogenesis.^28^^,^^29^ Recent medical treatments have been shown to be effective but bariatric surgery, specifically RYGB, remains one of the most effective methods for weight loss and resolution of obesity-related conditions, including MASLD.^30^^,^^31^ In this prospective cohort study and as expected, RYGB improved anthropometric and biochemical parameters as well as liver histology related to MASLD. In addition, we report findings showing that RYGB was associated with multi-omic alterations linked to specific liver histological changes, especially hepatic ballooning, which is required for the diagnosis of MASH, and NAS score. Hepatic gene expression showed upregulation of fatty acid β-oxidation (degradation), TCA cycle, and BCAA degradation while metabolomic profiling showed evidence of differential regulation of serum and faecal acylcarnitines and TCA/BCAA intermediate metabolites associated with alterations in intestinal microbiome. Together, these RYGB-associated changes may be involved in MASLD improvement and resolution post-RYGB (Fig. 9).

**Fig. 9:**
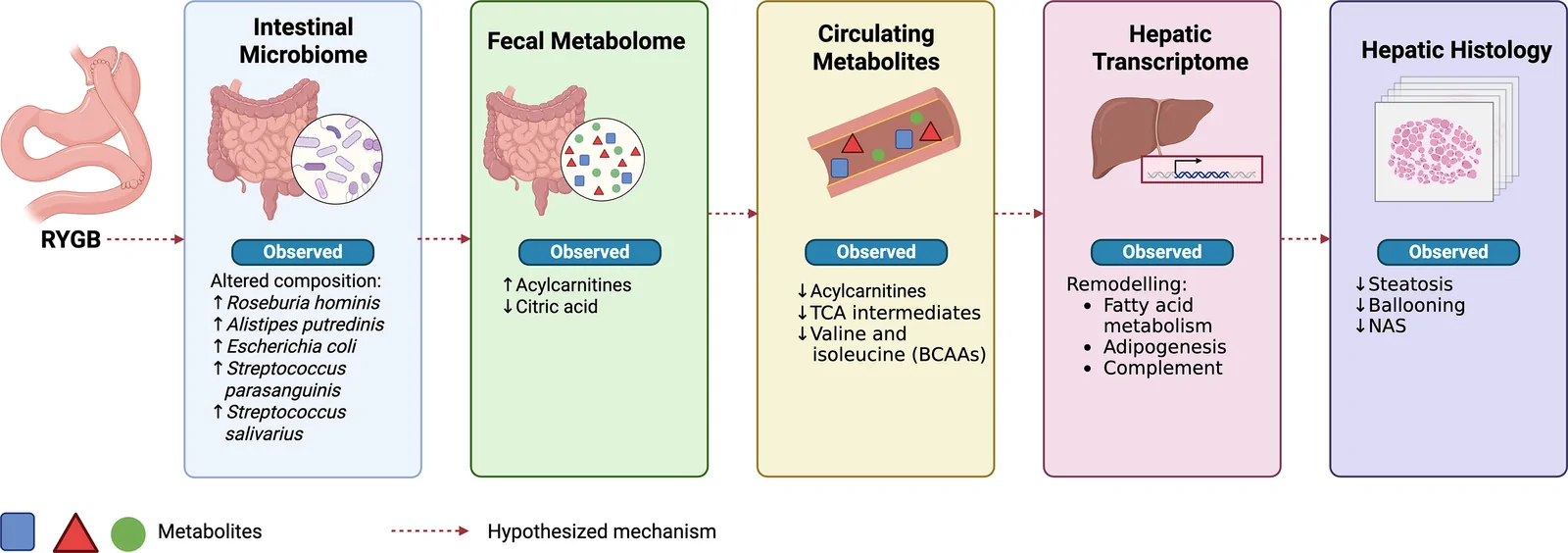
Hypothetical mechanism of RYGB-associated multi-omic changes along the gut–liver axis.

The pre- and post-RYGB liver samples were obtained using different biopsy modalities and this may have affected the relationship between changes in transcriptomic findings and changes in liver histology. Indeed, the patients had a liver wedge biopsy at the time of the RYGB surgery while a needle liver biopsy was performed at 12 months post-surgery. While this approach has been used in other similar studies,^12^^,^^14^ the biopsy modality could affect the findings due to sampling location. This is especially relevant given the spatial heterogeneity^32^^,^^33^ in features such as hepatic lipid metabolism, bile acid biosynthesis, glucose metabolism, immune response, signalling ligands and receptors which may affect results of transcriptomic analysis. There have been a few studies^32^^,^^33^ suggesting this potential effect. Using spatial transcriptomics, one study reported pericentral enrichment of genes and pathways related to lipid metabolism, bile acid synthesis, xenobiotic metabolism, and mitochondrial gluconeogenesis whereas periportal regions were enriched for immune-related and gluconeogenic gene expression.^32^ A similar lipid content bias was observed pericentrally in MASLD.^32^ This bias was further supported by a murine MASLD model which showed upregulation in pathways related to fatty acid elongation, lipoic metabolism, glycolysis and gluconeogenesis, and amino acid metabolism in central vein and pericentral regions.^33^ However, this literature was published after our study was completed. Since the biopsy type was linked to the timepoint, it could not be modelled as an independent co-variate because of the confounding effect of time post-RYGB. Consequently, this may have affected transcriptome changes due do spatial heterogeneity across the liver tissue and as a result, our study findings should be interpreted with this limitation in mind.

Generally, fatty acids cannot directly enter the inner membrane of the mitochondria where the oxidation and TCA cycle occurs.^34^ They are first metabolised into acyl-CoA which can then be converted into acylcarnitine by carnitine palmitoyltransferase 1 (CPT1; located on the outer mitochondrial membrane). Then, acylcarnitines are translocated to the inner mitochondrial membrane by carnitine-acylcarnitine translocase (CACT) where they are reconverted to acyl-CoAs by CPT2.^34^ Subsequently, acyl-CoAs undergo β-oxidation which sequentially shorten the acyl chain to generate acetyl-CoA.^34^ Under normal conditions, acetyl-CoA enters the TCA cycle to generate electron carriers (NADH and FADH_2_) which feed the electron transport chain for ATP synthesis (energy production) through oxidative phosphorylation.^34^^,^^35^ Under pathological condition, excess fatty acid flow to the mitochondria can exceed its oxidative capacity leading to incomplete β-oxidation and accumulation of partially oxidised intermediates (e.g. acylcarnitines).^34^ Initially, the TCA cycle activity may increase to accommodate excess acetyl-CoA; however, chronic fatty acid influx depletes electron acceptors (NAD^+^, FAD) required for the TCA cycle.^36^ In parallel, very-long-chain and branched-chain fatty acids are oxidised by the peroxisome which produces H_2_O_2_ and reactive oxygen species (ROS)^37^ leading to oxidative stress linked to ballooning of hepatocytes,^38^^,^^39^ a key feature of MASH. Our results suggest that RYGB is associated with increased fatty acid metabolism through peroxisomal and mitochondrial β-oxidation. While elevated peroxisomal β-oxidation may generate ROS and contribute to oxidative stress, the TCA cycle activity may also increase to accommodate the higher energy demand. This RYGB-associated metabolic response may support energy production but may also exacerbate oxidative stress and hepatic injury.

This concept is supported by our multi-omics approach. Our hepatic transcriptome analysis between pre- and 12 months post-RYGB revealed a distinct module of highly co-expressed genes which were involved in several metabolic and inflammatory pathways implicated in obesity and MASLD. Among these, fatty acid metabolism in this module was one of the pathways correlated with change in NAS and showed the strongest association with it. Therefore, we focused on this pathway for the downstream analysis. Overall, genes related to fatty acid metabolism hallmark pathway (e.g. ACADVL, ACOX1, EHHADH, SUCLG2, etc) were not only involved in mitochondrial and peroxisomal fatty acid β-oxidation but also TCA cycle and BCAA degradation, suggesting RYGB-associated remodelling of metabolism in the liver. Consistently, murine studies also reported RYGB-associated hepatic transcriptomic upregulation of pathways related to lipid metabolism and adipogenesis as well as downregulation of genes responsible for regulating lipolysis and producing triglyceride-rich lipoproteins.^40^^,^^41^

In terms of liver histology, upregulation in β-oxidation genes was positively correlated with changes in NAS, ballooning, and inflammation, indicating that stronger upregulation of these genes post-RYGB was linked to less pronounced histological improvement. This may be attributed to the RYGB-associated metabolic burden during rapid weight loss as the liver mobilises and processes the stored lipids.^42^ While post-RYGB weight loss can reduce oxidative stress and resolve MASLD and MASH in the majority of patients, newly developed or worsened MASLD and cirrhosis have been reported in a small group of patients.^43^^,^^44^ In a case series of 10 patients with worsened MASLD post-bariatric surgery (either RYGB or one-anastomosis gastric bypass), 8 patients required lengthening of the alimentary/common limb to improve the symptoms, 1 patient required liver transplantation and 1 patient died as a result of decompensated liver and septic shock.^43^ To support this, a meta-analysis of more than 3000 patients who underwent RYGB found newly developed or worsened MASLD features including inflammation and fibrosis in a subset of patients.^45^ Additionally, increase in ACOX1 gene post-RYGB suggests a potential upregulation in peroxisomal activity which may generate ROS and therefore, potentially exacerbate oxidative stress.^46^ These findings suggest a role for monitoring the liver function post-RYGB, because in a subset of patients, dysregulated fatty acid β-oxidation may impede liver improvement.

In addition, the RYGB-associated increase in fatty acid transport (from lipolysis) to the liver may exceed the mitochondrial oxidative capacity, leading to increase in TCA cycle activity.^36^ Consistent with this, we found that RYGB was associated with an increase in TCA cycle and membrane-associated genes (SUCLG2, SERINC1, and SDHC) which were negatively correlated with changes in ballooning of hepatocytes (a key indicator of MASH), suggesting a potentially beneficial role in improving hepatic injury. To the best of our knowledge, this has never been reported previously in humans with MASLD. However, in rats post-RYGB, peripheral TCA cycle intermediates were lower than in the portal vein.^47^ This suggests that an increase in TCA cycle activity post-RYGB may have reduced circulating TCA metabolites and potentially improved the mitochondrial function and ultimately, MASLD.^47^ However, liver transcriptome and histology were not assessed.^47^ In another murine model, genes involved in TCA cycle increased 9 weeks post-RYGB in the white adipose tissue, although hepatic genes were not reported.^48^ However, we could not determine which cell type contributed to the upregulation of genes related to the TCA cycle. Future studies using single-cell RNASeq or spatial transcriptomics would be required to determine whether these changes are observed in hepatocytes, immune cells or other hepatic cell types.

Overall, research on the direct contribution of the 10 genes enriched from fatty acid metabolism hallmark pathway in obesity and MASLD is limited. However, in the non-MASLD context, deficiency or reduction in expression of these genes could lead to lipid accumulation, inflammation and mitochondrial dysfunction in other tissues.^49^, ^50^, ^51^ One study in patients with rheumatoid arthritis found that SUCLG2 repression in CD4 T Cells led to accumulation of acetyl-CoA and subsequent proinflammatory and arthritogenic T cells.^50^ Another study in patients with lung adenocarcinoma reported that lung adenocarcinoma tissue had higher levels of SUCLG2 protein compared to the control.^51^ However, deletion of SUCLG2 could also be detrimental, and increase succinylation of mitochondrial proteins (a post-translational modification) leading to impairment or inhibition of the mitochondrial enzyme.^51^ In the context of bariatric surgery, a recent study of 26 patients with obesity (including 14 with MASH) who underwent RYGB found no significant alteration in these 10 genes in the liver despite improved lipid profile.^12^ However, their population was younger and had lower pre-RYGB BMI compared to ours. More mechanistic studies are needed to investigate the role of these genes in the context of RYGB, liver histology and MASLD.

We also examined whether the upregulation in fatty acid metabolism genes was accompanied by alterations in serum acylcarnitines as well as TCA- and BCAA-related metabolites. Acylcarnitines are fatty acid metabolites conjugated to carnitines for transport into the mitochondrial matrix^34^ where they participate in cellular energy pathways and undergo β-oxidation in the mitochondria.^34^ Given that RYGB improved metabolic profile, specifically the lipid profile, we investigated its impact on the above metabolites in the serum. Overall, circulating acylcarnitines decreased significantly post-RYGB. Supporting this, a bariatric surgery study found that fasting plasma medium- and long-chain acylcarnitines were significantly decreased at 12 months post-RYGB compared to the pre-surgical levels despite a transient increase at 2 months post-RYGB.^52^ Similarly, other bariatric studies reported reduction in circulating medium- and long-chain acylcarnitines post-bariatric surgery.^53^, ^54^, ^55^, ^56^ In contrast, short chain acylcarnitines generally increased and remained elevated even after a year^53^^,^^56^^,^^57^ and BCAA-related plasma acylcarnitines (C3, and C5) decreased post-RYGB,^54^^,^^56^^,^^58^ whereas we only found C4:1 to be elevated at 12 months post-RYGB. Thus, reduction in serum metabolites such as acylcarnitines may indicate improved mitochondrial and peroxisomal β-oxidation post-RYGB and could be consistent with the transcriptomic induction of mitochondrial and peroxisomal oxidation genes. However, we did not measure the mitochondrial oxidative activity.

Interestingly, changes in several medium- and long-chain acylcarnitines correlated with the SUCLG2 gene which is involved in the TCA cycle. SUCLG2 encodes a subunit of succinyl-CoA synthetase which converts succinyl-CoA to succinate.^51^ This finding implies a potential link between enhanced TCA cycle activity post-RYGB and systemic clearance of fatty acid intermediates. However, circulating succinate did not increase post-RYGB, possibly reflecting rapid utilisation in the TCA cycle or contribution of other sources.^59^ Impaired acylcarnitine metabolism has been linked to insulin resistance, inflammation, MASLD, cirrhosis and hepatocellular carcinoma (reviewed in^34^). Consistent with this, we observed that reductions in serum acylcarnitines post-RYGB correlated with improvements in hepatocellular ballooning, a key histological feature linked to disease progression. Together, these results suggest that improved fatty acid oxidation could contribute to histological improvement post-RYGB.

Other factors may contribute to changes in serum acylcarnitine levels, such as diet and malabsorption. Theoretically, dietary counselling and improved dietary composition post-RYGB could potentially contribute to changes in circulating acylcarnitines.^60^ However, only carbohydrate intake decreased, and no significant change was observed in fat intake using self-reported 3-day food intake which may be due to underreporting in these questionnaires.^61^ This suggests that changes in dietary composition may be less likely to play a role in the reduction of circulating acylcarnitines post-RYGB.

Acetyl-CoA from fatty acid oxidation may undergo further metabolism in the TCA cycle to produce energy in the mitochondria.^34^ To sustain this cycle, anaplerosis (the process of replenishing TCA intermediates which are continuously withdrawn for biosynthetic pathways) is essential for maintaining metabolic balance. In MASLD, however, this process is disrupted leading to accumulation of intermediates such as citric acid.^62^, ^63^, ^64^, ^65^ We found that RYGB reduced serum citric acid, fumaric acid and α-ketoglutaric acid, all of which are part of the TCA cycle. In contrast, sleeve gastrectomy (a different type of bariatric surgery) with n = 15 showed an increase in citric acid and other TCA metabolites^66^ and a trend towards increase was shown 6 months post-RYGB in n = 8 patients.^67^ However, these studies had small sample size,^66^^,^^67^ low BMI,^66^ and lower age^67^ and did not assess liver histology.^66^^,^^67^ In another study both RYGB and sleeve gastrectomy induced a temporary increase in circulating citric acid at 3 and 6 months but it was not observed at 12 months post-surgery.^68^ Therefore, more research is needed on RYGB-associated changes in circulating TCA-related metabolites.

BCAAs may also be metabolised into acetyl-CoA and succinyl-CoA which enter TCA cycle for further metabolism.^69^ Elevated BCAA has been observed in MASLD and obesity.^68^^,^^70^ In the context of bariatric surgery, we found significant reduction in serum BCAA valine and isoleucine post-RYGB. There was also a reduction in median leucine, but it did not reach significance. Our findings are consistent with other studies showing lower BCAA concentration post-bariatric surgery.^68^^,^^70^^,^^71^ Together, these findings suggest that RYGB may be associated with improved BCAA catabolism, contributing to the overall metabolic changes observed post-RYGB.

Reduced circulatory acylcarnitines and other metabolites may also reflect reduced intestinal absorption and greater delivery of acylcarnitines to the colon due to RYGB-induced anatomical changes post-RYGB leading to altered nutrient handling.^72^ We observed that the majority of the faecal acylcarnitines increased significantly post-RYGB while faecal citric acid decreased significantly. We have previously reported increases in faecal C5:1 and C16:1 acylcarnitines in mice receiving post-RYGB faecal samples compared to pre-RYGB faecal samples from human patients.^73^ This increase may impact the intestinal microbiome. Furthermore, some bacteria may metabolise acylcarnitines and TCA metabolites as a carbon and energy source.^26^^,^^27^^,^^74^ Carnitine and acylcarnitine availability have been associated with growth of *E. coli* and other members of Enterobacteriaceae, and carnitine can act as an osmoprotectant by helping the bacteria in maintaining osmotic balance under stress conditions in its environment.^26^^,^^74^ TCA cycle's citric acid is metabolised by diverse bacteria, including lactic acid bacteria and pathogens.^75^^,^^76^ For instance, *Vibrio cholerae*, the cholera-causing bacterium, uses citrate as sole carbon source, anaerobically, and competes with other bacteria for utilising citrate in mice.^76^ Interestingly, we found an increase in the relative abundance of bacteria such as *R. hominis*, which has been linked to acylcarnitine dysregulation^77^^,^^78^ and correlated with the change in faecal acylcarnitines. This finding suggests that elevated faecal acylcarnitines post-RYGB may alter microbiome composition and promote expansion of bacteria capable of using this energy source. However, more research is needed, and this capability may be species or strain-specific.^26^ In turn, it is possible that the expansion in these bacteria post-RYGB may lead to more production of other metabolites such as short-chain fatty acids and indoles^79^ which are beneficial, leading to improvement in insulin resistance, systemic inflammation and energy homoeostasis. Therefore, this suggests a potential complex crosstalk along the gut–liver axis post-RYGB.

Multi-omic changes post-RYGB and their relationship with changes in liver histology may have important clinical implication in patients with obesity and MASLD. Acylcarnitines may be useful as a monitoring tool to assess liver health instead of performing invasive liver biopsies; however, this requires further investigation. They are already being used as a diagnostic tool for metabolic diseases.^80^ In addition, research suggests that serum acylcarnitine composition and abundance is different between those with normal liver, MASLD and hepatocellular carcinoma.^81^ Some studies have also been investigating whether supplementation with L-carnitine could improve MASLD and MASH (e.g. NCT03193125, NCT03319199, NCT03439917). Additionally, the genes involved in mitochondrial and peroxisomal β-oxidation could be targeted for treating MASLD.^82^

This study has several strengths. First, our study population's age, sex and BMI were similar to other studies investigating impact of RYGB.^83^ The beneficial effect of RYGB on obesity and obesity-related comorbidities such as type 2 diabetes, MASLD and hyperlipidaemia have been extensively reported in previous studies.^84^ Second, integration of the liver transcriptome, serum and faecal metabolomes, and intestinal microbiome data provides a comprehensive view of metabolic remodelling after RYGB. Third, our study used paired-liver biopsies collected pre- and 12 months post-RYGB allowing assessment of transcriptome and histological changes in the liver. Lastly, faecal and serum acylcarnitines provide information on both circulating and excretory role of these metabolites post-RYGB.

Study limitations include its observational design and the fact that associations are not proof of causality. In addition, there is the potential effect of the different biopsy modality for pre-and post-RYGB liver biopsy. Future studies should use matched biopsy modality to validate these findings. Upregulation of the 10 core enrichment genes was not confirmed by qPCR due to limited tissue availability. Bulk RNASeq does not provide cell-specific resolution. Therefore, we could not determine in which hepatic cell type the observed gene expression changes occurred. We did not find associations between steatosis and core enrichment genes likely due to high proportion of patients with low steatosis grade prior to RYGB which may have impacted the effect size. In addition, our cohort was predominantly female, typical of bariatric surgery population.^85^^,^^86^ However, sensitivity analysis showed some of the key associations were maintained in the female population. Finally, RYGB alters the gastrointestinal anatomy, bypassing the duodenum and most of jejunum resulting in both dietary restriction and malabsorption.^87^ Thus, distinguishing between the changes arising from the weight-loss effect and the anatomical changes is not permissible. While our network analysis suggests that these alterations lead to potential improvement in liver histology, particularly the ballooning of hepatocytes, we acknowledge that causality cannot be established, and the directionality of some associations remains speculative. Stratified analysis of high versus low weight loss responder groups showed no significant difference in change in expression of the ten core enrichment genes. After adjustment for covariates, some key associations remained significant or were near significance. However, the findings from the regression analyses should be interpreted cautiously given the limited sample size. It is also unclear if these omic changes persist in the longer term. Future studies should address these limitations.

In conclusion, RYGB was associated with multi-omic changes in hepatic transcriptome, faecal microbiome and metabolome related to metabolic improvement. In the liver, some of these post-RYGB changes were associated with upregulation of genes involved in fatty acid metabolism and improvements in liver histology. However, the proposed gut–liver axis relationships require further validation with larger cohorts and functional experimental models to clarify these relationships and identify therapeutic targets for MASLD.

## Contributors

Conceptualisation- Katherine JP Schwenger, Johane P Allard, and Yasaman Ghorbani; Data curation - Yasaman Ghorbani, Katherine JP Schwenger, Elena M Comelli, Sandra E Fischer, Allan Okrainec, and Timothy Jackson; Funding Acquisition—Johane P Allard; Formal analysis–Yasaman Ghorbani, Katherine JP Schwenger, Heather Maughan, Anastasia Teterina, and Wendy Lou; Methodology–Katherine JP Schwenger, Johane P Allard; Writing (original draft)–Katherine JP Schwenger, Yasaman Ghorbani, and Johane P Allard; Writing (review and editing)—all authors. Katherine JP Schwenger, Johane P Allard, and Yasaman Ghorbani accessed and verified the data. All authors read and approved the final version of this manuscript.

## Data sharing statement

De-identified microbiome sequencing data is available to researchers upon reasonable request to the corresponding author upon request (Katherine.schwenger@uhn.ca) and senior author (dr.johane.allard@uhn.ca), subject to approval of a short proposal and a signed data use agreement to ensure patient privacy. The raw and processed transcriptomic data will be deposited into GEO repository once the ongoing publications are completed. Due to patient privacy, the underlying clinical data can only be requested from the corresponding author or the senior author.

## Declaration of interests

Author AO vice president of Canadian Association of Bariatric Physicians and Surgeons. He receives honoraria from Medtronic and J&J for speaking and teaching and has equity interest in GT Metabolic Solutions. EMC has received research support from Ocean Spray Cranberries and Lallemand Health Solutions and held contracts with Inquis Clinical Research. All are outside of this study. EMC held the Lawson Family Chair in Microbiome Nutrition Research at the University of Toronto and received funding from the Canadian Institutes of Health Research (CIHR), the Natural Sciences and Engineering Research Council of Canada (NSERC), the Banting and Best Diabetes Centre University of Toronto, the Joannah and Brian Lawson Centre for Child Nutrition University of Toronto. Other authors declare that they have no competing interests.

## Appendix A Supplementary data

Supplementary Workbook
