# Supplementary material for: Multi-omic profiling and pathways related to changes in liver histology after Roux-en-Y gastric bypass: a longitudinal study
Source: eBioMedicine. 2026 Sep 30;132:106498. doi: 10.1016/j.ebiom.2026.106498 (PMC13653980; doi:10.1016/j.ebiom.2026.106498)
Supplement: Supplementary file 1 [file mmc2.docx]

**Supplementary Materials**

**Table S1:** Anthropometric, biochemical and histological characteristics pre- and 12 months post-RYGB (n=38).

| **Variable** | **n** | **Baseline** | **n** | **12 months post-RYGB** | **p-value** |
| --- | --- | --- | --- | --- | --- |
| **Age (years)** | 38 | 50 (43, 55) | - | - | NA |
| **Sex (n, %)** | 38 | 35 (92.1%) | - | - | NA |
| **Females** |  | 35 (92.1%) |  |  |  |
| **Males** |  | 3 (7.9%) |  |  |  |
| **Weight (Kg)** | 38 | 131 (108, 146) | 38 | 91 (79, 101) | **<0.001** |
| **BMI (Kg/m^2^)** | 38 | 47.4 (43.0, 53.2) | 38 | 34.0 (29.8, 36.4) | **<0.001** |
| **Waist Circumference (cm)** | 32 | 135 (123, 146) | 29 | 106 (97, 112) | **<0.001** |
| **Systolic BP (mmHg)** | 37 | 127 (119, 136) | 32 | 119 (108, 125) | **<0.001** |
| **Diastolic BP (mmHg)** | 37 | 82 (80, 87) | 32 | 76 (71, 85) | **0.003** |
| **AST (U/L)** | 29 | 24 (19, 31) | 36 | 22 (17, 26) | 0.501 |
| **ALT (U/L)** | 35 | 25 (20, 42) | 38 | 22 (17, 28) | **0.013** |
| **ALP (U/L)** | 30 | 82 (71, 98) | 38 | 79 (65, 103) | 0.885 |
| **Glucose (mmol/L)** | 37 | 5.9 (5.3, 6.7) | 37 | 5 (4.7, 5.5) | **<0.001** |
| **Insulin (pmol/L)** | 28 | 117 (86, 193) | 37 | 44 (36, 56) | **<0.001** |
| **HOMA** | 28 | 4.86 (3.96, 9.48) | 37 | 1.74 (1.30, 2.07) | **<0.001** |
| **Presence of IR** | 28 | 23 (82%) | 37 | 5 (14%) | **<0.001** |
| **HbA1c** | 35 | 0.06 (0.06, 0.07) | 37 | 0.05 (0.05, 0.06) | **<0.001** |
| **Cholesterol (mmol/L)** | 34 | 4.36 (3.79, 5.03) | 37 | 4.13 (3.55, 4.78) | 0.059 |
| **TG (mmol/L)** | 34 | 1.37 (1.14, 1.76) | 37 | 1.01 (0.74, 1.30) | **<0.001** |
| **LDL (mmol/L)** | 34 | 2.53 (1.87, 3.16) | 37 | 2.18 (1.81, 2.58) | **0.009** |
| **HDL (mmol/L)** | 34 | 1.27 (0.97, 1.43) | 37 | 1.56 (1.38, 1.76) | **<0.001** |
| **Liver Disease** | 38 |  | 38 |  | **<0.001** |
| *Normal Liver* |  | 2 (5.3%) |  | 30 (79%) |  |
| *Simple Steatosis* |  | 22 (58%) |  | 7 (18%) |  |
| *MASH* |  | 14 (37%) |  | 1 (2.6%) |  |
| **Steatosis (%)** | 38 | 22.5 (7.5, 35.0) | 38 | 0 (0, 2.5) | **<0.001** |
| **Steatosis Grade** | 38 |  | 38 |  |  |
| *0* |  | 2 (5.3%) |  | 30 (79%) |  |
| *1* |  | 22 (58%) |  | 8 (21%) |  |
| *2* |  | 12 (32%) |  | 0 (0%) |  |
| *3* |  | 2 (5.3%) |  | 0 (0%) |  |
| **Ballooning of Hepatocytes** | 38 | 14 (37%) | 38 | 1 (2.6%) | **<0.001** |
| **Fibrosis** | 38 |  | 38 |  | 0.059 |
| *Stage 0* |  | 13 (34%) |  | 21 (55%) |  |
| *Stage 1–2* |  | 20 (53%) |  | 15 (39%) |  |
| *Stage 3–4* |  | 5 (13%) |  | 2 (5.3%) |  |
| **Lobular Inflammation** | 38 |  | 38 |  | **<0.001** |
| *0* |  | 14 (37%) |  | 32 (84%) |  |
| *1* |  | 14 (37%) |  | 5 (13%) |  |
| *2* |  | 10 (26%) |  | 1 (2.6%) |  |
| **NAS** | 38 |  | 38 |  | **<0.001** |
| *0* |  | 2 (5.3%) |  | 28 (74%) |  |
| *1–2* |  | 15 (39%) |  | 9 (24%) |  |
| *3–4* |  | 15 (39%) |  | 1 (2.6%) |  |
| *5+* |  | 6 (16%) |  | 0 (0%) |  |

Data presented as median (interquartile; IQR) or n (%), as appropriate. p-values were calculated using the paired Wilcoxon signed-rank test, McNemar, McNemar–Bowker, or Stuart–Maxwell Marginal Homogeneity tests, as appropriate.

ALP, alkaline phosphatase; ALT, alanine transaminase; AST, aspartate aminotransferase; BMI, body mass index; BP, blood pressure; HbA1c, haemoglobin A1c; HDL, high-density lipoprotein; HOMA, homeostatic model of assessment for insulin resistance; IR, insulin resistance; LDL, low-density lipoprotein; MASH, metabolic dysfunction-associated steatotic liver disease; NAFLD activity score; NL, normal liver; TG, triglyceride.

**Figure S1:** Quality control plots before and after normalization of gene expression data (n=38 patients). (A) MA plot before normalization; (B) MA plot after normalization with log-ratios centred around zero; (C) density plot of logCPM values before normalization; (D) density plot after normalization and; (E) boxplot of average expression per sample. 0 = pre-RYGB, 1 = 12 months post-RYGB.

**Supplementary Table S2:** Correlation between NAS and hallmark pathways identified in ME4 and ME5 (n=38 patients).

| **Hallmark** | **Histology** | **cor** | **p value** |
| --- | --- | --- | --- |
| HALLMARK_XENOBIOTIC_METABOLISM | NAS | 0.21 | 0.212 |
| HALLMARK_COAGULATION | NAS | 0.34 | **0.038** |
| HALLMARK_COMPLEMENT | NAS | 0.22 | 0.194 |
| HALLMARK_FATTY_ACID_METABOLISM | NAS | 0.38 | **0.019** |
| HALLMARK_ADIPOGENESIS | NAS | 0.33 | **0.042** |
| HALLMARK_BILE_ACID_METABOLISM | NAS | 0.21 | 0.202 |
| HALLMARK_EPITHELIAL_MESENCHYMAL_TRANSITION | NAS | 0.27 | 0.101 |
| HALLMARK_PEROXISOME | NAS | 0.28 | 0.089 |
| HALLMARK_OXIDATIVE_PHOSPHORYLATION | NAS | 0.30 | 0.071 |
| HALLMARK_HEME_METABOLISM | NAS | 0.24 | 0.148 |
| HALLMARK_INFLAMMATORY_RESPONSE | NAS | 0.09 | 0.577 |
| HALLMARK_MTORC1_SIGNALING | NAS | -0.04 | 0.808 |
| HALLMARK_XENOBIOTIC_METABOLISM | NAS | 0.21 | 0.212 |

Significant values (Spearman correlation, p<0.05) are shown in bold.

**Supplementary Table S3:** Comparison of serum acylcarnitines pre- versus 12 months post-RYGB (n=38 patients).

| **Serum Metabolite (umol/L)** | **Baseline (n=38)** | **12-monts post RYGB (n=38)** | **q-value** | **Direction** |
| --- | --- | --- | --- | --- |
| Serum C4:1 | 0.0536 (0.0499, 0.0640) | 0.0739 (0.0607, 0.0879) | **0.0047** | **↑** |
| Serum C4 | 0.2191 (0.1783, 0.2646) | 0.1915 (0.1528, 0.2218) | **0.0294** | **↓** |
| Serum C5 | 0.1423 (0.1048, 0.1733) | 0.0865 (0.0714, 0.1113) | **0.0001** | **↓** |
| Serum C4OH | 0.0705 (0.0466, 0.0923) | 0.0465 (0.0404, 0.0580) | **0.0108** | **↓** |
| Serum C6:1 | 0.0370 (0.0330, 0.0413) | 0.0313 (0.0298, 0.0339) | **0.0001** | **↓** |
| Serum C6 | 0.0680 (0.0614, 0.0865) | 0.0614 (0.0539, 0.0757) | **0.0056** | **↓** |
| Serum C5:1DC | 0.0197 (0.0174, 0.0215) | 0.0177 (0.0150, 0.0205) | **0.0486** | **↓** |
| Serum C8 | 0.2426 (0.2054, 0.2625) | 0.2198 (0.1924, 0.2752) | **0.0294** | **↓** |
| Serum C9 | 0.0329 (0.0288, 0.0419) | 0.0276 (0.0246, 0.0339) | **0.0215** | **↓** |
| Serum C7DC | 0.0381 (0.0336, 0.0432) | 0.0321 (0.0251, 0.0353) | **0.0003** | **↓** |
| Serum C10:2 | 0.0775 (0.0679, 0.0934) | 0.0543 (0.0499, 0.0593) | **0.0001** | **↓** |
| Serum C10:1 | 0.2710 (0.2508, 0.2925) | 0.2490 (0.2255, 0.2724) | **0.0048** | **↓** |
| Serum C10 | 0.2899 (0.2335, 0.3379) | 0.2446 (0.1895, 0.3507) | **0.0269** | **↓** |
| Serum C12:1 | 0.1677 (0.1462, 0.1925) | 0.1369 (0.1265, 0.1542) | **0.0001** | **↓** |
| Serum C12 | 0.0919 (0.0746, 0.1058) | 0.0649 (0.0608, 0.0739) | **0.0001** | **↓** |
| Serum C14:2 | 0.0436 (0.0378, 0.0581) | 0.0355 (0.0302, 0.0438) | **0.0015** | **↓** |
| Serum C14:1 | 0.1347 (0.1071, 0.1558) | 0.0934 (0.0728, 0.1148) | **0.0002** | **↓** |
| Serum C14 | 0.0576 (0.0487, 0.0682) | 0.0458 (0.0406, 0.0510) | **0.0001** | **↓** |
| Serum C14:1OH | 0.0127 (0.0106, 0.0145) | 0.0115 (0.0101, 0.0126) | **0.0221** | **↓** |
| Serum C16:2 | 0.0102 (0.0094, 0.0123) | 0.0089 (0.0084, 0.0093) | **0.0002** | **↓** |
| Serum C16:1 | 0.0515 (0.0471, 0.0590) | 0.0450 (0.0391, 0.0534) | **0.0294** | **↓** |

Data shown in median (Q1, Q3). Significant values (paired Wilcoxon signed-rank test; Benjamini–Hochberg adjusted, q<0.05) are shown in bold.

**Supplementary Table S4:** Comparison of serum metabolites of citrate cycle pre- versus 12 months post-RYGB (n=38 patients).

| **Serum Metabolite (umol/L)** | **Baseline (n=38)** | **12-monts post RYGB (n=38)** | **q-value** | **Direction** |
| --- | --- | --- | --- | --- |
| Succinic acid | 3.61 (3.34, 4.01) | 3.60 (3.38, 3.86) | 0.1558 | - |
| Citric acid | 123 (110, 135) | 112 (103, 127) | **0.0116** | **↓** |
| Fumaric acid | 0.96 (0.84, 1.32) | 0.79 (0.67, 1.01) | **0.0085** | **↓** |
| Alpha Ketoglutaric acid | 8.08 (7.35, 8.99) | 7.08 (6.11, 8.41) | **0.0016** | **↓** |
| Valine | 262 (228, 298) | 203 (189, 221) | **0.0009** | **↓** |
| Leucine | 137 (115, 148) | 123 (116, 132) | 0.1964 | - |
| Isoleucine | 64 (51, 72) | 56 (52, 60) | **0.0289** | **↓** |

Data shown in median (Q1, Q3). Significant values (paired Wilcoxon signed-rank test; Benjamini–Hochberg adjusted, q<0.05) are shown in bold.

**Supplementary Table S5:** Comparison of faecal acylcarnitines pre- versus 12 months post-RYGB (n=38 patients).

| **Faecal Metabolite (umol/g)** | **Baseline (n=38)** | **12-monts post RYGB (n=38)** | **q-value** | **Direction** |
| --- | --- | --- | --- | --- |
| Faecal C0 | 8.67e-03 (6.16e-03, 1.46e-02) | 5.3e-03 (3.46e-03, 8.21e-03) | **0.0006** | **↓** |
| Faecal C3:1 | 1.6e-04 (1.25e-04, 2.38e-04) | 1.16e-04 (9.2e-05, 1.48e-04) | **0.0084** | **↑** |
| Faecal C4:1 | 1.53e-04 (1.19e-04, 2.69e-04) | 2.09e-04 (1.75e-04, 5.29e-04) | **0.0104** | **↑** |
| Faecal C3OH | 1.65e-04 (1.34e-04, 2.02e-04) | 2.4e-04 (2.15e-04, 3.32e-04) | **0.0006** | **↑** |
| Faecal C5:1 | 1.82e-04 (1.49e-04, 2.88e-04) | 2.41e-04 (2.06e-04, 3.24e-04) | **0.0783** | **↑** |
| Faecal C5 | 1.36e-04 (1.11e-04, 1.9e-04) | 1.78e-04 (1.49e-04, 2.03e-04) | **0.0360** | **↑** |
| Faecal C5OH | 1.87e-04 (1.35e-04, 3.65e-04) | 1.4e-04 (1.18e-04, 1.67e-04) | **0.0009** | **↓** |
| Faecal C5:1DC | 9.81e-05 (6.78e-05, 2.06e-04) | 2.81e-04 (1.8e-04, 3.54e-04) | **0.0001** | **↑** |
| Faecal C5DC | 1.18e-04 (9.61e-05, 2.14e-04) | 3.88e-04 (3.3e-04, 6.36e-04) | **<0.0001** | **↑** |
| Faecal C8 | 1.18e-04 (8.26e-05, 1.9e-04) | 3.54e-04 (1.79e-04, 5.53e-04) | **<0.0001** | **↑** |
| Faecal C5MDC | 1.42e-04 (1.25e-04, 2.12e-04) | 5.22e-04 (3.1e-04, 6.23e-04) | **<0.0001** | **↑** |
| Faecal C9 | 1.4e-04 (1.03e-04, 2.43e-04) | 4.5e-04 (2.89e-04, 6.65e-04) | **<0.0001** | **↑** |
| Faecal C7DC | 1.31e-04 (9.22e-05, 2.58e-04) | 3.39e-04 (2.96e-04, 5.16e-04) | **<0.0001** | **↑** |
| Faecal C10:2 | 1.65e-04 (1.23e-04, 2.91e-04) | 7.32e-04 (4.22e-04, 1.26e-03) | **<0.0001** | **↑** |
| Faecal C10:1 | 3.34e-04 (2.24e-04, 6.54e-04) | 8.84e-04 (4.98e-04, 1.44e-03) | **<0.0001** | **↑** |
| Faecal C10 | 2.96e-04 (2.3e-04, 5.58e-04) | 1.21e-03 (8.61e-04, 1.54e-03) | **<0.0001** | **↑** |
| Faecal C12 | 2.57e-04 (2.17e-04, 3.57e-04) | 3.24e-04 (2.43e-04, 4.35e-04) | **0.0064** | **↑** |
| Faecal C14:2 | 7.27e-05 (5.72e-05, 9.69e-05) | 1.18e-04 (9.5e-05, 1.62e-04) | **0.0002** | **↑** |
| Faecal C14:1 | 4.33e-05 (2.57e-05, 8.45e-05) | 9.99e-05 (6.36e-05, 1.49e-04) | **0.0013** | **↑** |
| Faecal C14 | 1.29e-04 (1.02e-04, 1.94e-04) | 2.14e-04 (1.63e-04, 3.21e-04) | **<0.0001** | **↑** |
| Faecal C12DC | 1.13e-04 (8.99e-05, 1.82e-04) | 2.17e-04 (1.56e-04, 2.82e-04) | **<0.0001** | **↑** |
| Faecal C14:2OH | 8.63e-05 (6.74e-05, 1.09e-04) | 1.01e-04 (9.13e-05, 1.56e-04) | **0.0006** | **↑** |
| Faecal C14:1OH | 6.39e-05 (5.81e-05, 9.07e-05) | 1.07e-04 (8.83e-05, 1.7e-04) | **0.0002** | **↑** |
| Faecal C16:2 | 7.02e-05 (5.42e-05, 8.03e-05) | 7.85e-05 (6.63e-05, 1.04e-04) | **0.1068** | **↑** |
| Faecal C16:1 | 8.19e-05 (7.32e-05, 9.39e-05) | 1.06e-04 (8.3e-05, 1.26e-04) | **0.0267** | **↑** |
| Faecal C16 | 1.59e-04 (1.2e-04, 1.92e-04) | 2.01e-04 (1.69e-04, 2.8e-04) | **0.0003** | **↑** |
| Faecal C16:2OH | 5.91e-05 (5.3e-05, 7.51e-05) | 7e-05 (6.21e-05, 8.13e-05) | **0.0479** | **↑** |
| Faecal C18:2 | 1.57e-04 (1.3e-04, 1.8e-04) | 1.73e-04 (1.59e-04, 1.9e-04) | **0.0169** | **↑** |
| Faecal C18 | 1.06e-04 (8.38e-05, 1.38e-04) | 1.6e-04 (1.1e-04, 2.28e-04) | **0.0006** | **↑** |
| Faecal C18:1OH | 9.18e-05 (7.66e-05, 1.04e-04) | 1e-04 (8.89e-05, 1.12e-04) | **0.0036** | **↑** |

Data shown in median (Q1, Q3). Significant values (paired Wilcoxon signed-rank test; Benjamini–Hochberg adjusted, q<0.05) are shown in bold.

**Supplementary Table S6:** Comparison of faecal metabolites of citrate cycle pre- versus 12 months post-RYGB (n=38 patients).

| **Faecal Metabolites (umol/g)** | **Baseline (n=38)** | **12-monts post RYGB (n=38)** | **q-value** | **Direction** |
| --- | --- | --- | --- | --- |
| Succinic acid | 1.12e+00 (6.13e-01, 2.15e+00) | 7.9e-01 (4.85e-01, 1.45e+00) | 0.1221 | - |
| Citric acid | 3.91e-02 (2.03e-02, 5.02e-02) | 1.6e-02 (8.01e-03, 3.14e-02) | **0.0002** | **↓** |
| Fumaric acid | 1.2e-01 (7.91e-02, 2.75e-01) | 2.21e-01 (1.58e-01, 3.4e-01) | 0.2360 | - |
| alpha Ketoglutaric acid | 1.44e-01 (7.41e-02, 3.15e-01) | 2.46e-01 (1.28e-01, 4.08e-01) | 0.2360 | - |
| Valine | 2.91e+00 (1.91e+00, 3.82e+00) | 2.63e+00 (1.82e+00, 3.05e+00) | 0.2360 | - |
| Leucine | 2.75e+00 (2.03e+00, 3.67e+00) | 2.43e+00 (1.98e+00, 2.69e+00) | 0.2360 | - |
| Isoleucine | 1.46e+00 (9.51e-01, 2.11e+00) | 1.35e+00 (1.02e+00, 1.59e+00) | 0.2612 | - |

Data shown in median (Q1, Q3). Significant values (paired Wilcoxon signed-rank test; Benjamini–Hochberg adjusted,, q<0.05) are shown in bold.

**Supplementary Table S7:** Comparison of diet pre- versus 12 months post-RYGB (n=38 patients).

| **Characteristic** | **n** | **BL** | **n** | **12 months post-RYGB** | **p-value** |
| --- | --- | --- | --- | --- | --- |
| Energy (kcal) | 35 | 1629 (1274, 2048) | 24 | 1246 (1097, 1587) | 0.063 |
| Protein (g) | 35 | 67 (60, 96) | 24 | 68 (53, 101) | 0.656 |
| Carbohydrate (g) | 35 | 207 (149, 253) | 24 | 124 (107, 148) | **0.014** |
| Fat (g) | 35 | 63 (43, 84) | 24 | 56 (38, 80) | 0.702 |

Data shown in median (Q1, Q3). Significant values (paired Wilcoxon signed-rank test, p<0.05) are shown in bold.

**Supplementary Table S8:** Change in IM composition 12 months post-RYGB compared to baseline (n=38 patients).

| **Feature** | **coefficient** | **Standard Error** | **q value** |
| --- | --- | --- | --- |
| ***↓ Decreased post-RYGB*** | | | |
| *Anaerostipes hadrus* | -1.928 | 0.290 | **<0.0001** |
| *Bifidobacterium longum* | -1.913 | 0.455 | **0.0151** |
| *Clostridium innocuum* | -2.366 | 0.621 | **0.0220** |
| *Eggerthella lenta* | -2.055 | 0.408 | **0.0073** |
| *↑* ***Increased*** ***post-RYGB*** | | | |
| *Alistipes putredinis* | 1.961 | 0.498 | **0.0259** |
| *Escherichia coli* | 3.082 | 0.519 | **<0.001** |
| *Roseburia hominis* | 1.526 | 0.385 | **0.0197** |
| *Streptococcus parasanguinis* | 2.374 | 0.458 | **0.0008** |
| *Streptococcus salivarius* | 4.954 | 0.464 | **<0.0001** |

q-values were obtained from MaAsLin 3 mixed-effects models with timepoint as a fixed effect and subject as a random effect.

**Supplementary Table S9:** **Pairwise PLS latent-component correlations between changes in omic blocks (n=38 patients).** The blocks included change in ME4 genes, faecal metabolite, and bacterial species post-RYGB.

|  | **Component_1** | **Component_2** |
| --- | --- | --- |
| **Genes-Metabolites** | 0.75037724 | 0.74381906 |
| **Genes-Microbiome** | 0.87442891 | 0.9022821 |
| **Metabolites-Microbiome** | 0.98045981 | 0.84002172 |

**Supplementary Table S10: Comparison of change in the 10 core enrichment genes between low and high weight loss responders** (n=38 patients)**.**

| **Gene** | **High**  N = 19 | **Low**  N = 19 | **p-value** | **q-value** |
| --- | --- | --- | --- | --- |
| ALDH1A1 | 60.4109 (38.7814, 116.3833) | 42.6093 (6.9418, 87.4589) | 0.163 | 0.908 |
| ACADVL | -15.5900 (-30.0530, -5.6431) | -23.5934 (-31.4506, 5.3997) | 0.840 | 0.908 |
| EHHADH | 4.2523 (-16.7942, 18.8286) | -6.8332 (-17.7354, 10.9525) | 0.370 | 0.908 |
| SUCLG2 | 8.8325 (2.5865, 18.8888) | 2.6104 (0.9302, 13.4444) | 0.339 | 0.908 |
| SERINC1 | 5.8244 (-2.2031, 12.4419) | 3.3188 (-3.1051, 10.2675) | 0.488 | 0.908 |
| ALDH3A2 | 6.2297 (-0.1076, 13.7811) | 7.6702 (-2.2319, 18.3868) | 0.563 | 0.908 |
| ALDH9A1 | 10.6935 (5.3747, 17.4868) | 9.7884 (3.0353, 13.6792) | 0.665 | 0.908 |
| ACOX1 | 1.4230 (0.0169, 8.4491) | 4.3700 (-1.6993, 8.3717) | 0.908 | 0.908 |
| RETSAT | 4.3596 (-1.3916, 6.5174) | 3.8293 (-2.1071, 9.1474) | 0.817 | 0.908 |
| SDHC | 1.2530 (0.0237, 1.8221) | 0.7335 (-0.6178, 1.4894) | 0.212 | 0.908 |

Data shown in median (Q1, Q3). Significant values (Wilcoxon rank-sum test; Benjamini–Hochberg adjusted, q<0.05) are shown in bold

**Supplementary Figure S2: Circle Correlation Plots for pairwise PLS models (n=38 patients).** Only top 25 features for each dimension are displayed, subsetting by those with a correlation above 0.5.

**Figure S3: Sample plots from the DIABLO model according to NAS resolution status (n=38 patients).** Plots show the distribution of participants across the A) component 1 and component 2; and B) component 3 and component 4 for each omics block, including ME4 genes, faecal metabolites, and bacterial species.

A)

**Figure S4: Heatmap of change score of selected multi-omic features from DIABLO in A) component 1, b) component 2, C) component 3 and; D) component 4** (n=38 patients)**.** Features were selected by the fourth DIABLO component from the genes from ME4 module, faecal metabolites, and bacterial species selected. Rows represent participants grouped by NAS resolution status, and columns represent selected features from each omics block.

A)

B)

**C)**

**D)**

**References:**

[1]	Brunt EM, Janney CG, Di Bisceglie AM, Neuschwander-Tetri BA, Bacon BR. Nonalcoholic steatohepatitis: a proposal for grading and staging the histological lesions. Am J Gastroenterol 1999;94:2467-2474.

[2]	Rinella ME, Lazarus JV, Ratziu V, Francque SM, Sanyal AJ, Kanwal F, et al. A multisociety Delphi consensus statement on new fatty liver disease nomenclature. Hepatology 2023;78:1966-1986.
